# Revolutionizing ovarian cancer therapy by drug repositioning for accelerated and cost-effective treatments

**DOI:** 10.3389/fonc.2024.1514120

**Published:** 2025-01-14

**Authors:** Edgar Yebran Villegas-Vazquez, Francisco Pável Marín-Carrasco, Octavio Daniel Reyes-Hernández, Andrea S. Báez-González, Lilia Patricia Bustamante-Montes, Teresita Padilla-Benavides, Laura Itzel Quintas-Granados, Gabriela Figueroa-González

**Affiliations:** ^1^ Laboratorio de Farmacogenética, UMIEZ, Facultad de Estudios Superiores Zaragoza, Universidad Nacional Autónoma de México, Ciudad de México, Mexico; ^2^ Department of Molecular Biology and Biochemistry, Wesleyan University, Middletown, CT, United States; ^3^ Coordinación de Investigación, Centro Universitario siglo XXI, Estado de México, Toluca, Mexico; ^4^ Colegio de Ciencias y Humanidades, Plantel Cuautepec, Universidad Autónoma de la Ciudad de México, Ciudad de México, Mexico

**Keywords:** cancer, ovarian cancer, conventional treatment, drug repositioning, cancer hallmarks

## Abstract

Drug repositioning, the practice of identifying novel applications for existing drugs beyond their originally intended medical indications, stands as a transformative strategy revolutionizing pharmaceutical productivity. In contrast to conventional drug development approaches, this innovative method has proven to be exceptionally effective. This is particularly relevant for cancer therapy, where the demand for groundbreaking treatments continues to grow. This review focuses on drug repositioning for ovarian cancer treatment, showcasing a comprehensive exploration grounded in thorough *in vitro* experiments across diverse cancer cell lines, which are validated through preclinical *in vivo* models. These insights not only shed light on the efficacy of these drugs but also expand in potential synergies with other pharmaceutical agents, favoring the development of cost-effective treatments for cancer patients.

## Introduction

**Figure 1:**
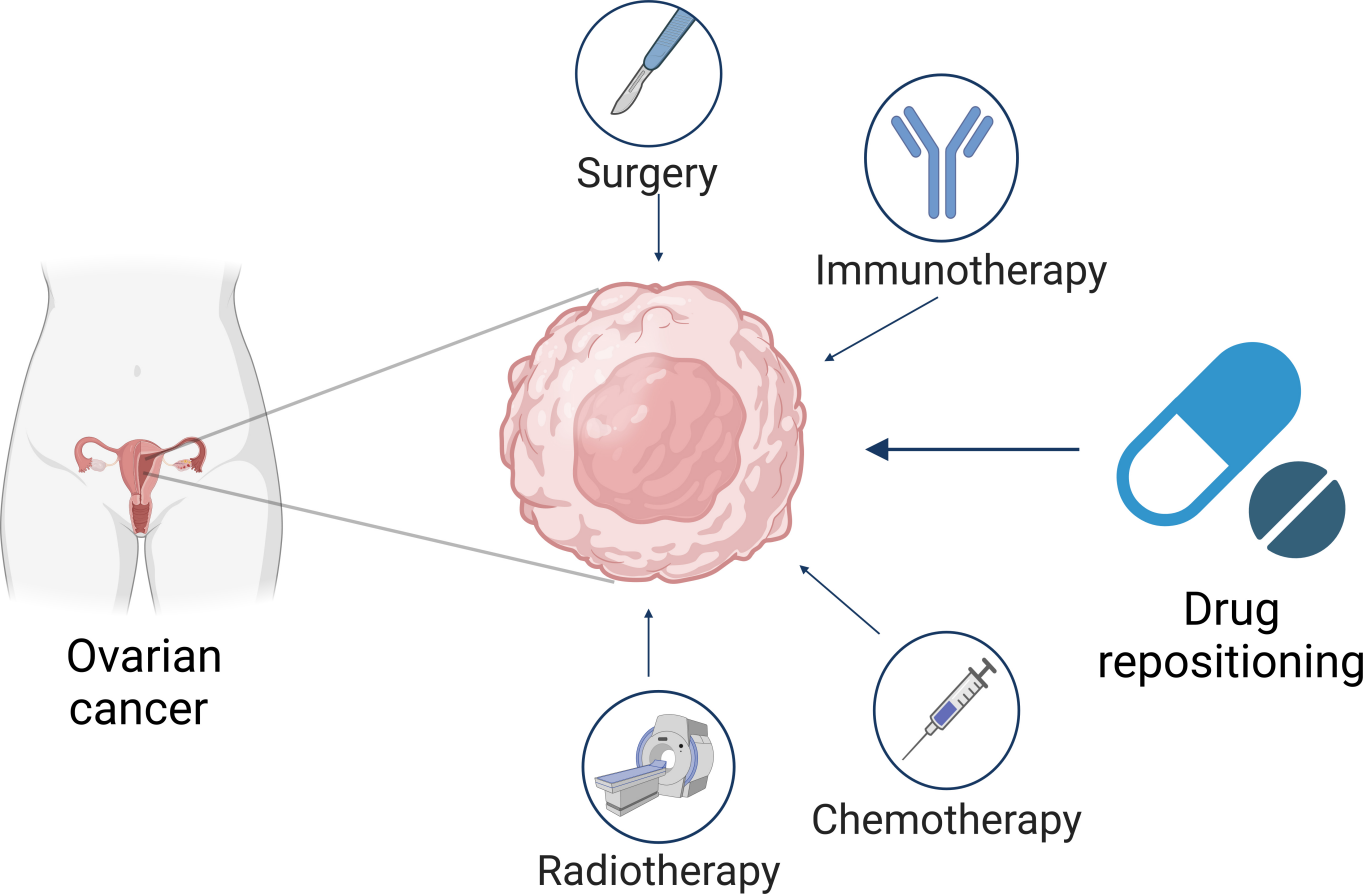
Schematic representation of ovarian cancer treatments. This diagram illustrates the general approach to ovarian cancer therapy. Outline the importance of drug repositioning besides surgery, immunotherapy, chemotherapy, and radiotherapy (Figure created with Biorender).

Drug repositioning, also known as drug repurposing, is a strategy that involves identifying new therapeutic uses for existing drugs beyond their original indications. This approach has gained the attention of the scientific community due to its potential to expedite the drug development process, reduce costs, and maximize the utility of existing pharmaceutical agents (**Figure 1**). Drug repositioning possesses multiple advantages, as it presents increased efficiency by shortening the drug development timeline, as existing drugs have already endured various stages of testing for safety and efficacy. It capitalizes on the existing safety, toxicity, and pharmacokinetic data of approved drugs, significantly reducing the time and financial investment compared to *de novo* drug discovery. This approach has been especially valuable in addressing unmet medical needs, such as rare diseases and conditions lacking effective treatments. Thus, repurposing of drugs often results in a more cost-effective process than developing entirely new compounds, as it bypasses the extensive research and development phases, as repositioned drugs usually have established safety profiles, minimizing the risks associated with introducing entirely new substances. In fact, in recent years, the introduction of new drugs to the market has seen a decline owing to the adverse outcomes witnessed in medical trials and challenges in pharmacokinetics (1). However, significant progress in computational sciences, including bioinformatics, machine learning and computational chemistry, coupled with advancements in -omics sciences and high-throughput screening technologies, has enabled the exploration of drugs with multiple target molecules. These have broadened their potential applications and pharmacological benefits (2).

Drug repurposing has multiple applications in cancer treatment. For instance, repositioned drugs offer the opportunity to target specific pathways or mechanisms relevant to cancer, potentially introducing alternative treatment options. In addition, this strategy allows the identification of synergies between repositioned drugs and existing cancer treatments, which can lead to the development of more effective combination therapies. However, there are limitations associated with drug repurposing, such as patent protection for the original application of the drugs, which may pose challenges for repositioning efforts. Ultimately, administration of repurposed drugs in patients may also require adjustments and further investigation of the pharmacological conditions through *in vitro, in vivo* and clinical studies. Thus, designing appropriate clinical trials for repositioned drugs requires careful consideration of the new therapeutic context and side effects, as well as dosing regimens and possible coadjuvant treatments must also be considered when treating patients. Despite these limitations, since the safety and pharmacokinetic parameters of many drugs are well known, the increasing interest in drug repositioning is helping to determine new favorable outcomes of these drugs. Emerging approaches, such as molecular docking studies and other computer-aided methods, are helping to model novel ligand-targeting strategies and to help drug repositioning take a better landscape in cancer therapy and unlocking the potential of already existed drugs.

In addition, combination therapies that integrate repurposed drugs with existing pharmaceutical agents hold significant promise for enhancing treatment efficacy in cancer and other malignancies. These synergies can target multiple pathways simultaneously, overcoming limitations such as drug resistance and heterogeneity within tumors, as discussed in specific cases below. However, the complexity of drug-drug interactions in combination therapies poses challenges. In this regard, additional limitations of drug repurposing combined with existing therapies include variations in pharmacokinetics, off-target effects, and potential antagonistic interactions. Moreover, the tumor microenvironment, patient-specific genetic factors, and dosage optimization add further layers of complexity. Thus, in order to utilize this strategy in the clinic, it is essential to perform rigorous preclinical and clinical studies, to avoid adverse effects in patients.

This review offers a comprehensive analysis of drug repositioning in ovarian cancer (OC) therapy, synthesizing findings from *in vitro* and preclinical models while also acknowledging the limited data from clinical studies. Despite inherent limitations, the contributions of these models are invaluable for advancing our understanding of potential treatments. *In vitro* studies, particularly with cancer cell lines, continue to be essential tools in screening and identifying promising drug candidates. Although these models cannot fully mimic the complexities of human tumors, they provide controlled environments that allow for detailed investigation of drug effects on cancer cell biology, offering critical insights into drug responses, mechanisms of action, and preliminary efficacy (3–5). These findings can then be validated through more sophisticated *in vivo* models. Preclinical mouse models, for example, provide vital data on the pharmacokinetics, pharmacodynamics, and toxicity of repositioned drugs. These models allow for a comprehensive evaluation of drug responses within the context of tumor microenvironments, immune responses, and metabolism, even though they do not perfectly replicate human disease (6–8). Preclinical models thus serve as an important bridge between *in vitro* assays and clinical applications, often informing the design of clinical trials. Clinical translation remains a significant challenge, but the few studies conducted on repurposed drugs for OC in patients have demonstrated encouraging results. These trials, although limited in scale, provide critical insights into the pharmacokinetic properties, dosing regimens, and side effect profiles of repositioned drugs, underscoring the importance of further research to confirm their clinical efficacy. However, the current focus on short-term experimental models limits our understanding of the long-term therapeutic benefits and safety of these drugs. Longitudinal clinical studies are crucial to fully evaluate their sustained effectiveness and potential side effects, paving the way for the future of OC therapy.

## Ovarian cancer

Epithelial ovarian cancer is the rapid growth of cells with abnormal function and structure with the potential to invade and destroy other healthy tissues (9). Among gynecological cancers, OC has a superior mortality rate because of its difficult early diagnosis resulting widely metastatic within the abdomen (10), placing OC as the 3^rd^ most common gynecological cancer around the world in 2020 (11). Low- and middle-income countries presented the highest mortality rates of OC but its incidence was highest in high-income countries (12).

The nomenclature for OC subclassification includes five main histological types: high-grade serous (HGSOC), low-grade serous (LGSOC), endometrioid (ENOC), clear cell (CCOC), and mucinous (MOC) (13–15). HGSOC tumors are solid masses of cells characterized by slit-like fenestrations and structured with papillary, glandular or cribriform architecture (16). LGSOC tumors are distinguished by a monotonous proliferation of cuboidal, low columnar cells, mild to moderate atypia without nuclear pleomorphism, a mitotic index reaching up to 12 mitoses per 10 high-power fields (HPF) and invasion (17–19). Histopathological distinction of ENOC tumors from HGSOC is challenging, but the use of some discriminatory immunohistochemistry tools such as Wilms’ tumor 1 (WT1) lead to better tumor classification. ENOC tumors generally lack WT1 expression, whereas HGSOC tumors overwhelmingly exhibit WT1 positivity. In addition, ENOC is positive for the estrogen receptor (ER) in ≥75% of cases and for the progesterone receptor (PR) in over 60% of cases, with the majority (80%) of patients being also positive for wild-type Tumor Protein p53 (*TP53*) (20–23). Immunohistochemical markers, including WT1, Napsin A, hepatocyte nuclear factor-1-beta (HNF-1β), ER, and PR, are employed to distinguish CCOC from HGSOC and ENOC tumors. CCOC tumors are WT1 negative, Napsin A/HNF-1β positive, and EP/PR negative (22–24). MOC tumors are large, unilateral mucinous growths that negative for the WT1 and Napsin A markers, and approximately 60% of cases express a mutant version of p53 (23, 25).

**Figure 2:**
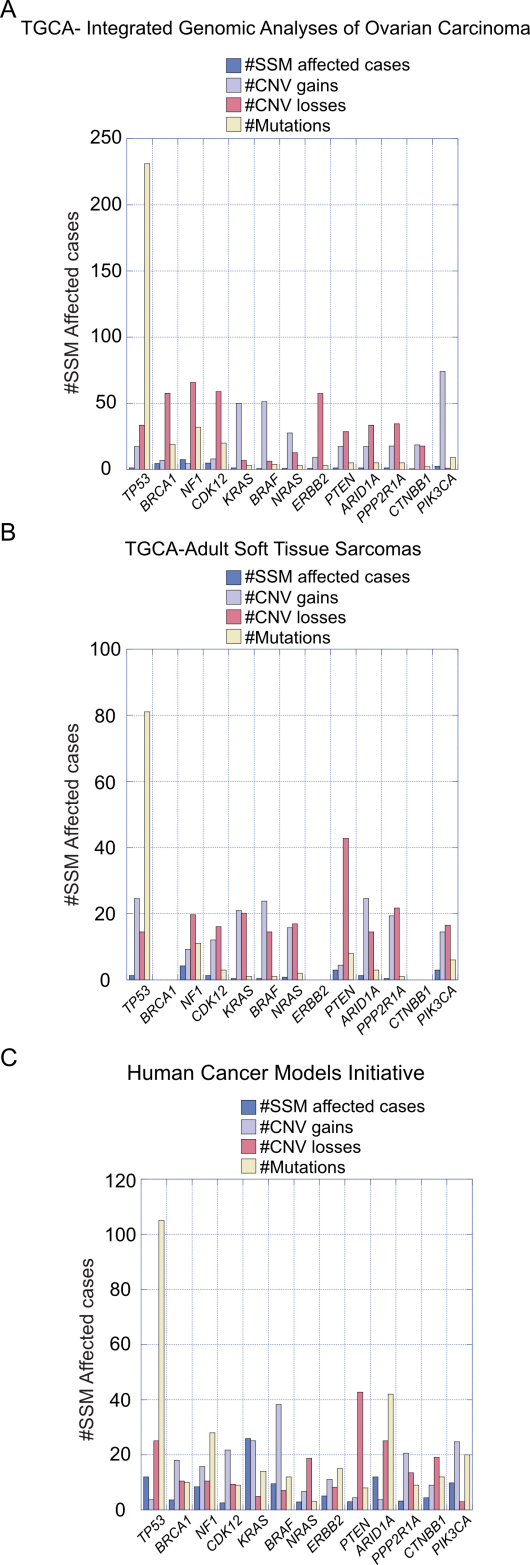
Recorded percentage of cases with mutations in specified genes relevant to ovarian carcinoma development. **(A)** Data presented are from the Integrated Genomic Analyses of Ovarian Carcinoma and **(B)** from the Comprehensive and Integrated Genomic Characterization of Adult Soft Tissue Sarcomas, sourced from The Cancer Genome Atlas (TCGA). **(C)** Information on relevant genes was obtained from the Human Cancer Models Initiative (HCMI) Cancer Model Development Center.

Ovarian tumors are histopathologically heterogeneous, resulting genetic mutations specific for each epithelial OC subtype, which can be used as targets of treatment (10). For instance, near-ubiquitous mutations of p53, Breast Cancer Gene 1/2 (*BRCA1/2*), Neurofibromin 1 (*NF1*), and Cyclin-Dependent Kinase 12 (*CDK12*) are characteristic of HGSOC subtype (10, 26). LGSOC subtype is characterized by mutations in Kirsten Rat Sarcoma Viral Oncogene Homolog (*KRAS*), B-Raf Proto-Oncogene, Serine/Threonine Kinase (*BRAF*), Neuroblastoma RAS Viral Oncogene Homolog (*NRAS*), and Erb-B2 Receptor Tyrosine Kinase 2 (*ERBB2*, also known as HER2) mutations, while Phosphatidylinositol-4,5-Bisphosphate 3-Kinase Catalytic Subunit α (PI3KCA), Phosphatase and Tensin Homolog (*PTEN*), AT-Rich Interaction Domain 1A (*ARID1A*), and Protein Phosphatase 2 Scaffold Subunit α (*PPP2R1A*) mutations are present in ENOC subtypes. Moreover, mutations in *PI3KCA*, *ARID1A*, Catenin β1 (*CTNNB1*), *PTEN*, and *PP2R1A* are also found in the CCOC subtype. Finally, the MOC subtype is characterized by *KRAS* and *ERBB2* mutations (10). Changes in DNA methylation patterns also contribute to the development of OC, being the hypermethylation of the *BRCA* promoter a common example detected in 15-30% of patients (27–30). **Figure 2** shows the recorded percentage of cases where mutations on the genes outlined here, as reported by the Integrated Genomic Analyses of Ovarian Carcinoma (**Figure 2A**) and the Comprehensive and Integrated Genomic Characterization of Adult Soft Tissue Sarcomas (**Figure 2B**) from The Cancer Genome Atlas (TGCA) Human Cancer Models Initiative (HCMI) Cancer Model Development Center (**Figure 2C**). This information highlights the relevance of the presence of the mutations described above in the development of OC.

**Figure 3:**
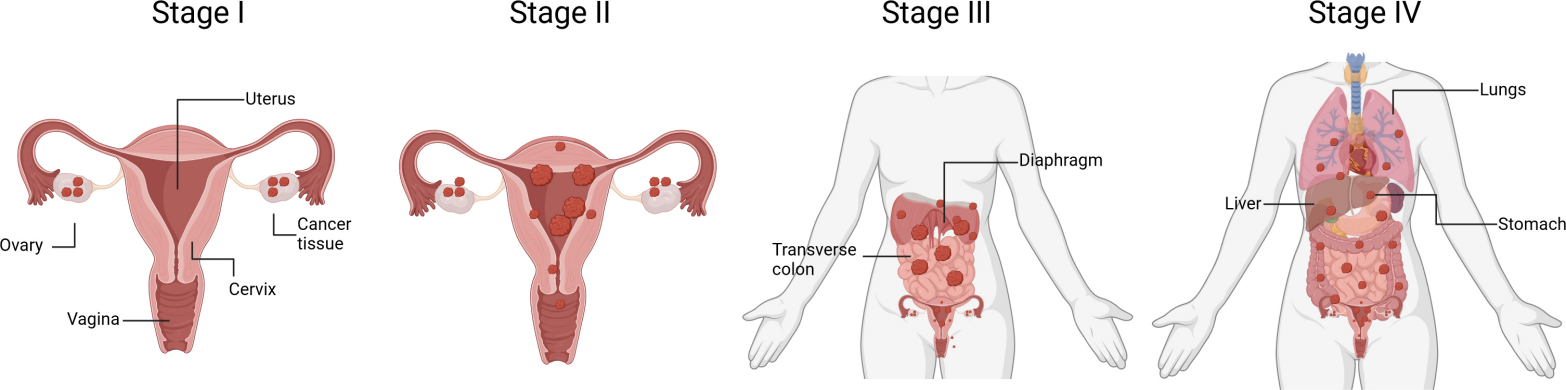
Schematic representation of ovarian cancer progression. The diagram outlines the four stages of OC, ranging from Stage I to Stage IV. Stage I signifies cancer that is confined to one or both ovaries. Stage II indicates cancer that has spread beyond the ovaries but is still within the pelvis. Stage III represents cancer that has advanced beyond the pelvis and has spread to the abdominal lining or nearby lymph nodes. Stage IV denotes cancer that has metastasized to distant organs beyond the abdominal cavity, such as the liver or lungs. Understanding the stage of OC is crucial for determining treatment options and predicting prognosis. (Figure created with Biorender).

Ovarian cancer staging is determined by the severity of the disease, considering factors such as tumor size, spread to nearby tissues, and the presence of distant metastasis (**Figure 3**). The International Federation of Gynecology and Obstetrics (FIGO) system is commonly used for ovarian cancer staging. Stage I is when cancer is confined to one or both ovaries, being sub-stage IA limited to one ovary, no tumor on the external surface; no ascites (fluid in the peritoneal cavity) present containing malignant cells, and substage IB when both ovaries are affected but no tumor is found on the external surfaces; no ascites containing malignant cells. Stage II is when cancerous tissue is found in one or both ovaries with pelvic extension. Substage IIA includes an extension to the uterus and/or fallopian tubes with no evident tumor on the external surfaces and neither ascites containing malignant cells. Substage IIB is when malignant tissue is extended to other pelvic tissues, but no tumor is found on the external surfaces and there are still no ascites containing malignant cells. Stage III OC involves one or both ovaries, the tumor may be spread to the peritoneum outside the pelvis and/or metastasis to the retroperitoneal lymph nodes and/or the omentum. Patients in stage III can be further classified in three substages according to the severity of metastasis. For instance, substage IIIA is determined by microscopic peritoneal metastasis beyond the pelvis; substage IIIB involves macroscopic peritoneal metastasis beyond the pelvis less than 2 cm in size; and substage IIIC includes abdominal metastasis greater than 2 cm in size and/or positive retroperitoneal lymph nodes. Finally, stage IV is diagnosed once malignant cancer cells have spread to distant organs or tissues, being predominant in the pleural fluid (substage IVA) and in the parenchyma of the liver and other distant organs (Substage IVB) (31–33).

## Etiology of ovarian cancer

Ovarian cancer is a complex disease with multifactorial etiology, and its development involves a combination of genetic, hormonal, and environmental factors. Risk increases with age and genetic factors such as *BRCA* mutations (34, 35). Mutations in essential genes for DNA repair, such as MutL Homolog 1 (*MLH1*) and MutS Homolog 2 (*MSH2*), which are also predominant in Lynch syndrome patients, also increase the risk of ovarian cancer (36). Having descendants at an early or advanced age, nulliparity, and absence of full-term pregnancy are classic risk factors of OC (37). Long-term use of estrogen-only hormone replacement therapy, without progesterone, has also been linked to an increased risk of OC (38). Recently, an increase between 7 and 28% of OC incidence in women ranging from 15-40 years of age has been detected, likely due to the “normalization” of unhealthy lifestyles such as overweight and obesity (12). In addition, social determinants included the human development index and highest gross domestic product per capita and lifestyle characteristics such as physical inactivity, alcohol use, and prevalence of smoking (39). Some studies have suggested a potential link between the use of talcum powder in the genital area and an increased risk of OC as well (40). Pre-existing conditions such as lipid disorders, hypertension, diabetes, estrogen exposure, and metabolic syndrome are also associated with high OC incidence rates (12, 41–46). Evidence has also pointed to immune responses as potential contributors to OC, such as general dysregulation of the immune system and impaired immune surveillance (47), as well as chronic inflammation in the pelvic region (48).

OC incidence varies by race and ethnicity, with differences observed in the rates of diagnosis and outcomes, which in many instances correlate with the socioeconomic background. White females are the largest population affected, with 14.1 cases per 100,000 women. Then the next highest ethnicity incidence is in (Hispanic females, with a rate of 9.8 affected women per 100,000 individuals. This is followed by Asian/Pacific Islanders, African Americans, and American Indian/Alaska natives, whose incident rates are 9.0, 8.5, and 7.9 patients, respectively, per 100,000 women (49–51). Mortality rates are also dependent on different racial and ethnic groups. African American women often experience lower survival rates compared to non-Hispanic white women. This is largely due to limited access to healthcare and socioeconomic factors, which contribute to these important health disparities. The age at diagnosis and access to screening are also a relevant concern for appropriate treatment and survival, as the age at which OC is diagnosed may also vary by race and ethnicity. Some studies suggest that African American women may be diagnosed at a younger age compared to non-Hispanic white women (52–54).

## Prognosis of ovarian cancer

Different histological subtypes of OC may have distinct prognoses. For example, HGSOC, the most common subtype, tends to be more aggressive. Overall, poor prognosis of OC is associated with the age and stage of disease at diagnosis (10, 55). Younger patients, particularly those diagnosed at premenopausal ages, may have a more favorable prognosis.; and the patient’s overall health and ability to tolerate treatment can also influence prognosis. In terms of disease stage, for stages I, II, III and IV, the 5-year relative survival rate after diagnosis is estimated at 89%, 70%, 36% and 17%, respectively; while the 10-year-relative survival rate was 84%, 59%, 23% and 8%, for those stages. In general, the overall relative survival rate at 2, 5 and 10 years after diagnosis was 65%, 44% and 36%, respectively (56). Thus, survival rate in women diagnosed at stage I in 5-year is 90% (55, 57). The five-year survival rate is about 80% in patients with disseminated disease to adjacent tissues. In metastatic patients the survival rate is 25% (55, 57). Unfortunately, the 5-year survival rate is less than 50% in patients diagnosed at an advanced OC stage (58–60). In 12-24 months, most patients relapse and die from progressive chemotherapy-resistant tumors (61).

## Current therapies for OC treatment

The first-line treatments for OC are surgery, radiotherapy, and chemotherapy. Surgery is performed to remove the tumor tissue in its entirety. Surgery is also useful for histopathological diagnosis and staging of the tumor, according to the International FIGO (10). Surgical procedures include bilateral salpingo-oophorectomy, tumor debulking, total hysterectomy, and omentectomy (62). In addition, different trials used preoperative chemotherapy when interval debulking surgery is performed. Trials reported a reduction in postoperative morbidity (63, 64). Therefore, no difference in survival was observed when a second surgical procedure to complete tumor debulking (10).

For treatment of early-stage OC, cytotoxic chemotherapy improves survival (8%) (63, 64). Treatments with platinum (carboplatin or cisplatin) have been used as the first-line treatment (10). Cisplatin is a platinum-containing chemotherapy drug that is commonly used in the treatment of OC as it binds covalently to DNA in cancer cells, blocking replication and transcription (65, 66). Regimens with two cytotoxic agents improve survival, thus the standard treatment for OC is a combinatory therapy of paclitaxel or docetaxel with platinum-containing drugs (67–69). Rucaparib olaparib, niraparib, and talazoparib are poly(ADP-ribose) polymerase inhibitors (PARPi) that have been accepted by the FDA as chemotherapeutic drugs for OC treatment (70). However, platinum-containing drugs, paclitaxel, olaparib, niraparib, and bevacizumab, cause drug resistance in some OC patients (70).

Novel treatments for OC management employed several strategies (71) that include *i*) target morphomolecular OC types by using PARPi for HGSOC subtype, or the use of inhibitors of the Mitogen-activated protein kinase kinase (MEK) or aromatase inhibitors for LGSOC subtype (72, 73). *ii*) new clinical trial designs, such as umbrella and baskets studies (74, 75). *iii*) new inhibitors (ATRi) against the Ataxia telangiectasia and Rad-3 related kinase (ATR), such as prexasertib, adavosertib (76–82). *iv*) synergistic therapies combining drugs targeting both the tumor and its microenvironment, such as antiangiogenic compounds (e.g., bevacizumab, cediranib), immunotherapy, and chemotherapy (83–89). *v*) enhanced therapeutic delivery using antibody-directed conjugates or targeted radiotherapy (90–94). Among these, ATR serves as a promising target in cancer due to its role in signaling DNA lesions, replication stress, and regulating the S and G2/M checkpoints, offering potential for exploiting dysregulated DNA damage responses (95). Drugs that interfere with DNA repair, such as PARP inhibitors (Olaparib), are used particularly in patients with BRCA mutations (96).

Novel strategies to overcome drug resistance challenges against OC include the use of monoclonal antibodies. Specifically, the humanized IgG1 monoclonal antibody bevacizumab is directed against Vascular Endothelial Growth Factor (VEGF), and it is currently used for OC therapy (97). Immunotherapy is also an emerging strategy to overcome drug resistance. In this case, immune checkpoint inhibitors, such as pembrolizumab, are being investigated in clinical trials to boost the immune system to target cancer cells (98).

Treating OC presents significant challenges, including late diagnosis, chemoresistance, and the limited efficacy of available therapies. Thus, there is still a need to consider and develop alternative mechanisms to combat OC progression. In this regard, drug repositioning represents an excellent alternative therapy for patients who have developed drug resistance, which may result in failure to prevent recurrence, particularly in advanced stages. Moreover, the tumor microenvironment and genetic heterogeneity of OC further complicate treatment development. Drug repurposing offers a promising avenue to address these challenges by leveraging existing drugs with known safety profiles for new therapeutic uses. In consequence, these emerging approaches can expedite treatment availability and reduce development costs while exploring combinatorial strategies for enhanced efficacy​. Drug repurposing studies hold promise as a bridge to personalized medicine, improving outcomes for OC patients.

### Drug repositioning for OC treatment

**Figure 4:**
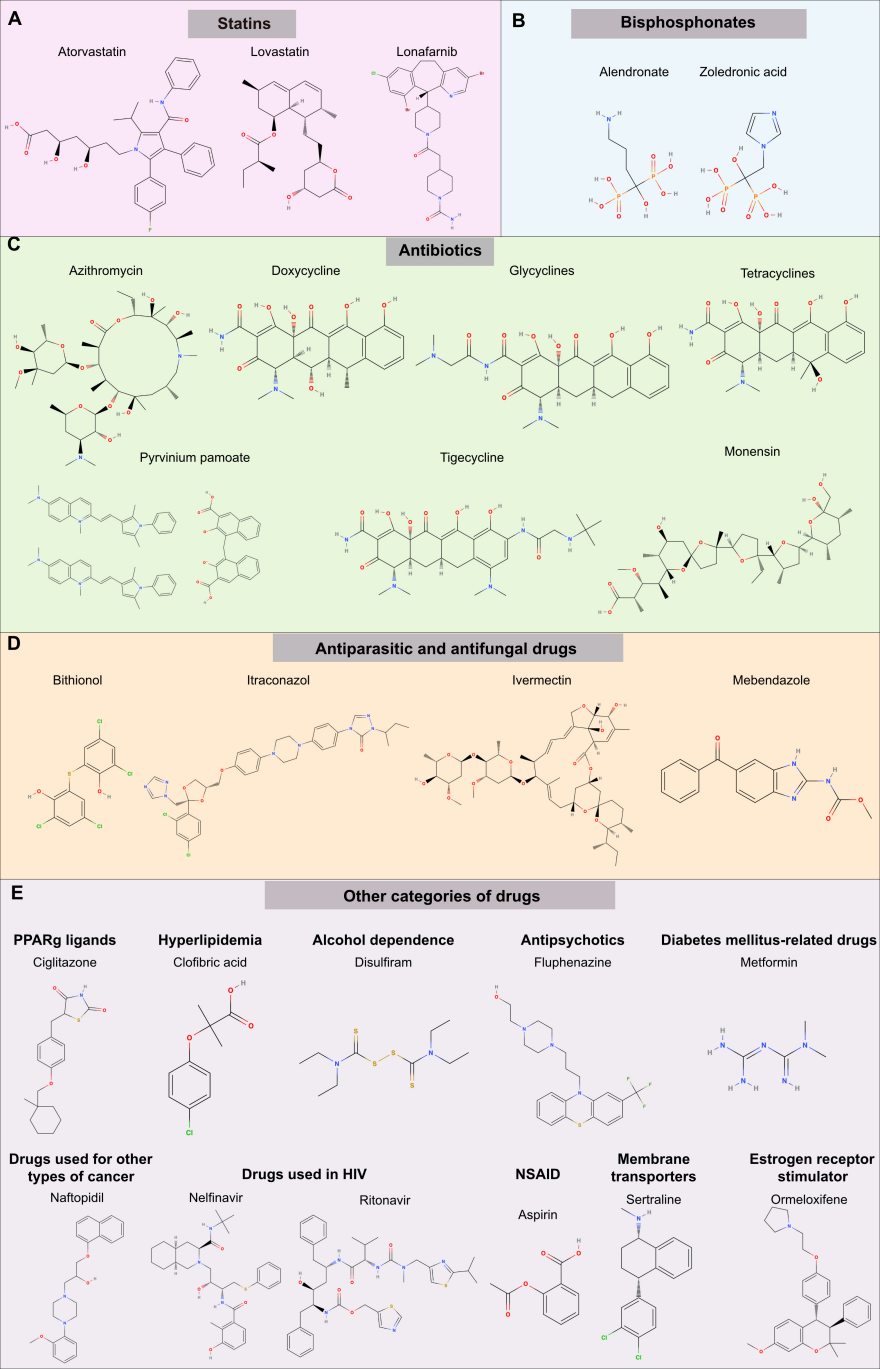
Chemical structure of potential drugs to be repurposed against ovarian cancer. The diagram illustrates potential drug candidates for repurposing in the treatment of OC. Various pharmaceutical agents target different pathways; see **Table 1** for a summary of mechanisms. The groups represented are **(A)** statins, **(B)** bisphosphonates, **(C)** antibiotics **(D)** antiparasitic and antifungal drugs, **(E)** other categories of drugs including PPARγ inhibitors, compounds used to treat hyperlipidemia, alcoholism, and mental disorders, diabetes, other types of cancer (prostate), HIV infection, Non-steroidal anti-inflammatory drugs (NSAIDs), membrane transporters and estrogen receptors. Each drug offers a unique mode of action that could potentially enhance therapeutic outcomes in OC management.

Amid the evolving landscape of OC treatment, the exploration of innovative therapeutic strategies becomes imperative. Drug repositioning, a promising approach, involves repurposing existing drugs to uncover novel and effective treatments for this disease. As indicated above, this strategy harnesses the potential of compounds already approved for other indications, accelerating the development of cost-effective and targeted therapeutic options. In this review, we provide insights into drug repositioning specifically for OC, exploring its challenges, successes, and transformative therapeutic impact. Main highlights and structure of the compounds presented here are summarized in **Table 1** and **Figure 4**.

**Table 1:** Drug repositioned for ovarian cancer treatment.

Drug	Original target	*In vivo/in vitro* study model	Mechanism of action or target molecule in OC treatment	Relevant hallmark involved	Concentration/Dosage	Ref
Atorvastatin	Cardiovascular diseases prevention	*In vitro* cell lines Hey and SKOV3	Inhibit the biosynthesis of the cholesterol enzyme mevalonate, inhibiting difosfatfarnesyl and diphosphategeranylgeranyl	Induction of apoptosis	1 μM, 50 μM, 150 μM	(102)
Lovastatin	Cholesterol treatment	*In vivo* cell lines SKOV-3, OVCAR-5 xenograft in mice	Synthesis disruption of acetyl Co-A in the endoplasmic reticulum	Growth suppression and apoptosis	12.5 mg/kg	(104)
Lonafarnib	Progeria treatment	*In vitro* cell lines SKOV-3, OVCAR-5	Inhibit the biosynthesis of the lipids of the RAS protein in the farnesyl chain for the structuring of the cell membrane	Induction of apoptosis	From 10 nM to10 µM	(108)
Alendronate	Osteoporosis treatment	*In vivo* xenograft cells SKOV3, OVCAR5 in mice models mogp-Tag	Inhibit cholesterol biosynthesis by blocking farnesyl pyrophosphate synthase	Induction of apoptosis	15 mg/kg	(108)
Zoledronic acid	Osteoporosis treatment	*In vitro* cell lines OVCAR-3 and MDAH-2774	Inhibit cholesterol biosynthesis by blocking farnesyl pyrophosphate synthase	Induction of apoptosis	5 µM	(115)
		*In vitro* extracted samples of ovarian tissue from human patients	Inhibit cholesterol biosynthesis by blocking farnesyl pyrophosphate synthase	Induction of apoptosis	From 2.2 to 69 μM	(117)
Azithromycin	Antibiotic	*In vitro* cell lines SKOV3, Tov21G, ES2	Inhibits protein synthesis by binding to the 30s ribosome subunit and inhibiting peptide translocation.	Deregulating cellular energetics, suppressing proliferation and induction of apoptosis	250 μM	(119)
Doxycycline	Antibiotic	*In vitro* cell lines Tov21G, ES2	Inhibits protein synthesis by binding to the 30S ribosomal subunit	Deregulating cellular energetics, suppressing proliferation and induction of apoptosis	50 μM	(119)
Glycylcyclines	Antibiotic	*In vitro* ovarian cancer cell lines SKOV3, Tov21G, ES2	Inhibits protein synthesis by binding to the 28s ribosome subunit and inhibiting peptide translocation.	Induction of apoptosis	From 10 nM to10 µM	(119)
Tetracycline	Antibiotic	*In vitro* ovarian cancer cell lines SKOV3, Tov21G, ES2	Binding to the subunit 28S small mitoribosome then inhibiting mitochondrial anabolism	Deregulating cellular energetics, suppressing proliferation and induction of apoptosis	From 1 μM to 250 μM	(119)
Pyrvinium pamoate	Anthelmintic	*In vitro* cell lines SKOV3, Tov21G, ES2	Suppression of the mitochondrial activity complex and dysregulation of STAT transcription over the Mito OXPHOS	Deregulating cellular energetics, suppressing proliferation and induction of apoptosis	250 nM, 500 nM	(119)
Tigecycline	Antibiotic	*In vitro* cell lines SKOV3, Tov21G, ES2	It inhibits protein transduction by binding to the 30S ribosomal subunit and blocking the entry of aminoacyl tRNA (transfer RNA) molecules into the ribosomal site	Deregulating cellular energetics, suppressing proliferation and induction of apoptosis	50 µM	(119)
Monensin	Antibiotic	*In vitro* cell lines SK-OV-3, A2780, OVCAR3, CAOV-3	Decrease in phosphorylated ERK and MEK proteins by activation of E-cadherin and claudin, participating in the epithelial-mesenchymal transition	Inhibition of growth and prevention of cell differentiation	0.2 µM, 1 µM, 5 µM	(61)
Bithionol	Antiparasitic and anthelmintic	*In vivo* xenograft cancer cells SKOV-3-luc-D3 in mice Foxn1	Dysregulation of the cell cycle by ROS generation and NF-kB inhibition	Induction of apoptosis	30 mg/kg, 60 mg/kg, 120 mg/kg, 240 mg/kg	(151)
Itraconazole	Antifungal	*In vivo* humans	Antiangiogenic function by inhibition of growth receptor 2 VEGFR2 and phosphorylation of ERK, hedgehog, and TOR pathways	Antiangiogenic and growth suppression	From 400 to 600 mg	(157)
		*In vivo* xenograft cancer cells in mice, endothelial human cancer cells	Antiangiogenic function by inhibition of growth receptor 2 VEGFR2 and phosphorylation of ERK, hedgehog, and TOR pathways	Antiangiogenic and growth suppression	From 100 to 600 mg	(156)
Ivermectin	Antiparasitic and anthelmintic	*In vitro* cell line SKOV-3	Induction of stop cell cycle in G0-G1 by modulation of growth factor proteins by kinase PAK-1 inhibiting	Growth suppression	5 µM	(163)
		*In vitro* cell lines TYK-nu, KOC7c, SKOV3 and MRUG-S	Induction of stop cell cycle in G0-G1 by modulation of growth factor proteins by kinase PAK-1 inhibiting	Growth suppression	5 µM, 20 µM	(160)
Mebendazole	Antiparasitic and anthelmintic	*In vivo* xenograft in mice ovarian cancer cell lines: MES-OV (p53 R282W), ES2 (p53 S241F), A2780 (p53 wild type), SKOV3 parental (p53 null)	Block tubulin polymerization that generates dysfunctional microtubules and difficult cytoskeleton structural functions	Deregulating cellular energetics, suppressing proliferation and induction of apoptosis	50 mg/kg	(172)
		*In vitro* lines OVCAR8CR and SKOV3CR	Inhibits the sign ways ELK/SRF, NFKB, MYC/MAX y E2F/DP1	Deregulating cellular energetics, suppressing proliferation and induction of apoptosis	From 0 µM to 4 µM	(176)
Ciglitazone	Type 2 diabetes, atherosclerosis	female nu/nu mice xenografted with subcutaneous OVCAR-3 tumors	Inhibits cell growth by causing cell cycle arrest and apoptosis in ovarian cancer cells. Reduce prostaglandin E2 (PGE2) in a COX-2-independent manner, induce apoptosis, inhibit angiogenesis, and inhibit tumor progression.	Inhibit cell cycle and tumor progression induction of apoptosis, inhibit angiogenesis	15 mg/kg intraperitoneally once a week	(183, 184, 275, 276)
Clofibric acid	Hyperlipidemia	OVCAR-3 cells and female BALB/c nu/nu mice model	Inducing carbonyl reductase, which promotes the conversion of PGE2 to PGF2α	Suppression of cell proliferation and increasing apoptosis	500 μmol/L	(190)
Disulfiram	Alcoholism treatment	*In vitro* cell lines OVCAR-3, SKOV3, OV-MZ-30, OV-MZ-31, OV-MZ-37, and OV-MZ-38	Irreversible structural cell damage through oxidized disulfide bonds in paranuclear proteins	Induction of apoptosis	From 0 µM to 5 µM	(196)
Fluphenazine	Antipsychotic	*In vitro* cell line OVCAR-3	Oligonucleosomal cleavage of genomic DNA and caspase substrate polyadenosine diphosphate ribose suggest induces caspase-dependent apoptotic cell death	Induction of apoptosis	3.84 µM	(202)
Metformin	Regulate the amount of sugar in the blood	*In vitro* cells Hey and SKOV3ip1	Inhibits mitochondrial complex I (NADH dehydrogenase) activity and cellular respiration of electrons transported by glucose deprivation, stopping Krebs cycle function	Induction of apoptosis	From 10 mM to 40 mM	(204)
Naftopidil	Prostatic hyperplasia treatment	*In vitro* cell lines IGROV1-R10, SKOV3	Increased expression of Bim, Puma, and Noxa proteins, which affect mitochondria and induce apoptosis	Induction of apoptosis	0 µM, 25µM, 50 µM	(214)
Nelfinavir	Antiretroviral	*In vitro* cell lines PEO1/PEO4/PEO6 y PEO14/PEO23	Inhibits phosphorylation in AKT and ERK, causing damage to nuclear DNA and endoplasmic reticulum	Suppress proliferation and induce apoptosis	From 1 µM to 50 µM	(215)
Ritonavir	Antiretroviral	*In vitro* cell lines MDH-2774 and SKOV-3	Apoptosis induction by phosphorylation of AKT that inhibits the PI3K/Akt	Antiangiogenic, growth suppression, and apoptosis	20 µM	(217–219)
Sertraline	Antidepressant	*In vivo*, cell lines OVCAR-8, human ovarian adenocarcinoma NCI-ADR/RES (NAR) xenografts in mice	Interferes with cellular pathways of TNF-MAP4K4-JNK, the antiapoptotic PI3K/Akt/mTOR, AMPK/mTOR axis, and TCTP/P53 feedback loop and with the cytosolic Ca2+ levels	Induction of apoptosis	1 µM	(227)
Ormeloxifene	Contraceptive	*In vitro* cell lines A2780, A2780-CP and SKOV-3	Phosphorylation of p53 and the Akt pathway, increasing cell cycle inhibitors p21 and p27 and inhibition of Bcl-xl on mitochondrial function, generating apoptosis	Growth suppression and apoptosis	10 µM, 20 µM	(256)
Thalidomide	Anti-emetic	*In vitro* SKOV-3 cells	Decreases the TNF-α, MMP-9 and MMP-2 secretion	Did not have a significant effect on cell proliferation and growth	(4×10^-7^ - 2×10^-5^ M)	(237)
Dasatinib	Philadelphia chromosome-positive acute lymphoblastic leukemia or chronic myeloid leukemia	SKOV-3 and Hey cells Hey xenograft model	Reduced the phosphorylation of AKT, mTOR, p70S6K, and S6 kinase expression.	Autophagic cell death	300 nM for SKOv3 cells and 150 nM for HEY cells	(243)
Imatinib	Chronic myelogenous leukemia and gastrointestinal stromal tumors	*In vitro* cell lines: C272-hTert/E7, C889/hTert, CSOC848, CSOC908, and CSOC918	Inhibits phosphorylation of PDGFRα and Akt in PDGFRα-specific manner	Inhibition growth and cell cycle progression in a PDGFRα-specific manner	IC50 < 1 μm	(246).

Among relevant chemical compounds being repurposed towards treatment of OC, statins are medications commonly prescribed to lower cholesterol levels in the blood. Statins inhibit an enzyme involved in the production of cholesterol in the liver. By reducing cholesterol levels, statins help lower the risk of cardiovascular events such as heart attacks and strokes. Strategies to inhibit the mevalonate pathway have also been applied to dyslipidemic diseases. Statins reduce the hydroxymethylglutaryl coenzyme A (HMG-CoA) reductase activity, which is enzymatically essential in the upstream part of the mevalonate pathway, resulting in a reduction in cholesterol levels in blood (99). Thus, considering the mechanism of action of statins related to mevalonate pathway inhibition, they are used to treat hypercholesterolemia (100). However, recent findings suggested that these molecules have antitumoral activities by causing apoptosis in tumor cells (101). For instance, atorvastatin (ATO) inhibits cell proliferation and invasion, while decreasing cell adhesion of cultured OC cells. Besides, ATO induces cellular stress, autophagy, apoptosis, and arrest cell cycle at G1 phase through Akt/mTOR pathway inhibition and MAPK pathway activation (102, 103). ATO also decreased the expression of *VEGF*, matrix-metalloproteinase-9 (*MMP9*), and the proto-oncogene cellular myelocytomatosis (*c-Myc*) in Hey and SKOV3 cultured OC cellular models (102). Experiments using the JQ1 selective inhibitor of bromodomain-containing proteins in Hey and SKOV3 OC cells also increased their sensitivity to the anti-proliferative activity of ATO (102). Another statin example that can be repurposed towards OC treatment is Lovastatin. This is another HMG-CoA reductase inhibitor that has been effective in reducing the proliferation of OC Hey and SKOV3 cells *in vitro* and *in vivo* murine models. Lovastatin delays tumorigenesis, proliferation, and cell cycle progression and suppresses tumor growth by influencing the cholesterol biosynthetic pathway (104, 105). Simvastatin, another HMG-CoA reductase inhibitor, reduces the production of cholesterol in the liver, thus lowering the levels of total cholesterol and low-density lipoprotein (LDL) cholesterol in the bloodstream. Simvastatin reduces the risk of cardiovascular events and has been shown to possess anti-metastatic and anti-tumorigenic effects in OC treatment. For instance, ID8, 28-2, and 30-2 cells treated with simvastatin had increased expression of apoptotic markers starting at 10 µM in 28-2 and 30-2 cells, and 50 µM for ID8 cell lines, suggesting that simvastatin induced cell death and decreased cell viability. Simvastatin was further shown to inhibit OC cell proliferation in a dose-dependent manner as measured by 3-[4,5-dimethylthiazol-2-yl]-2,5 diphenyl tetrazolium bromide (MTT) assay in Hey and SKOV3 OC cells (106, 107). Other inhibitors of mevalonate pathway have been shown to promote autophagic responses. Examples of this are 6-fluoromevalonate, YM-53601, lonafarnib, and GGTI-298. These compounds can induce the expression of autophagy biomarkers such as LC3A (human microtubule-associated protein 1 light chain 3 gene LC3A and LC3B (human microtubule-associated protein 1 light chain 3 gene LC3B) and inhibit cell proliferation in a dose-dependent manner (108). Specifically, Lonafarnib inhibits the enzyme farnesyltransferase, which plays an important role in post-translational modification of proteins. Its primary function is to add a farnesyl group to specific proteins, in a process known as farnesylation.

Lonafarnib also inhibits protein prenylation in the mevalonate pathway, inhibiting cell proliferation with higher efficiency than 6-fluoromevalonate and YM-53601 (108). Besides, lonafarnib induces the expression of *LC3A* and *LC3B* genes, suggesting that the activation of autophagy impairs cell proliferation (108).

Bisphosphonates, such as alendronate, are anti-osteoporotic drugs that also inhibit the mevalonate pathway. Bisphosphonates delayed tumor formation and decreased tumor cell proliferation in a murine model of OC (108). Alendronate inhibits proliferation in OC SKOV3 and chemoresistant OVCAR5 cell lines *in vitro*. It induces the expression of *LC3A* and *LC3B* genes, indicating autophagy activation. In a transgenic OC mouse model (mogp-TAg), alendronate reduces total tumor mass, suggesting suppression of tumor initiation, and implies a potential chemo-preventive effect in OC development (108). In addition, alendronate reduces Rho activation by inhibiting the mevalonate pathway, resulting in the inhibition of cell migration in Caov-3, OC cells (109). Furthermore, the alendronate treatment (1mg/kg/d) reduced the tumor burden by ∼88% in female nude mice (BALB-c *nu/nu*) injected with Caov-3 (110). Together, *in vitro* and *in vivo* evidence strongly suggested findings that alendronate had potential as a drug for OC treatment (108–110).

Zoledronic acid is another example of a bisphosphonate drug, mainly prescribed for bone-related conditions like osteoporosis and cancer-induced bone complications. This drug inhibits bone resorption, promoting bone strengthening (111, 112). Specifically, zoledronic acid inhibits the activity of osteoclasts, the cells responsible for breaking down bone tissue, helping to maintain bone density and strength. It has also been used for the treatment of multiple myeloma and metastatic breast cancer and to treat hypercalcemia (high levels of calcium in the blood) associated with malignancy (113, 114). Zoledronic acid has been used in combination with gossypol, a natural polyphenolic compound used as a male contraceptive and with demonstrated anticancer properties in prostate cancer and leukemia. Combined, these two drugs render a synergistic cytotoxic and apoptotic effect in OC OVCAR-3 and MDAH-2774 cell lines (115). This combined treatment repressed the transcriptional expression of angiogenic molecules such as the inhibitor of differentiation or DNA binding (ID-1), EPH (Ephrin) receptors B2 and B4 (EPHB2/B4), laminin α-5 (LAMA-5), the fibroblastic growth factor (FGF2) and FGF receptor-3 (FGFR3), midkine (MDK), thymidine phosphorylase (TP), platelet-derived growth factor A (PDGF-A), and the cytokine CXCL-1, which plays a pivotal role in angiogenesis (115). Furthermore, experiments where NCI-H929, OPM-2, U266 and RPMI-8226 myeloma cell lines were pre-treated with simvastatin and then combined with antimyeloma drugs resulted in the apoptotic cascade (116). The combination of fluvastatin with zoledronic acid enhanced the chemosensitivity to the ATP-based tumor assay (ATP-TCA) in twenty-two pre-treated (mostly with platinum-based chemotherapy) ovarian carcinomas. Sequential drug experiments showed that pretreatment of tumor cells dissociated from solid carcinomas with fluvastatin resulted in decreased sensitivity to zoledronic acid (117). Mechanistically, zoledronic acid and fluvastatin treatment enhance the effects that involved Ras prenylation. Thus, implying that prior to bisphosphonate administration, statins would be expected to block the entry of mevalonate into the pathway, reducing the substrate concentration for the step that is blocked by zoledronic acid, potentially enhancing the effectiveness of the combination (117).

Research has suggested cancer could be managed as an infectious disease, in other words, by taking advantage of antibiotics that inhibit mitochondrial biogenesis, which is essential for clonal expansion and survival of cancer stem cells (118). This idea arose from the anabolic nature of cancer stem cells, which require mitochondrial biogenesis for proliferation and survival (118). Thus, targeting mitochondrial biogenesis is an alternative avenue that might render anti-tumorigenic effects useful against cancer treatment. Examples of antibiotics that impair mitochondrial biogenesis as a side effect are pyrvinium pamoate, doxycycline, azithromycin, tigecycline, and chloramphenicol, which make these compounds potential candidates in the treatment of OC (119). Mechanistically, antibiotics such as erythromycin, chloramphenicol, tetracyclines, glycylcyclines and pyrvinium pamoate target three main mitochondrial molecules. These are the mitochondrial 39S/large and the 28S/small ribosome subunits and mitochondrial oxidative phosphorylation proteins (OXPHOS), such as complex I of the electron transport chain (119–121). Azithromycin was shown to inhibit the tumor-sphere formation of OC SKOV3, ES2 and Tov21G cells, demonstrating the potential for cancer management (119). Conventionally, doxycycline has been used in the treatment of prostatitis, urinary tract infections and acne due to its anti-inflammatory properties (122–124). Doxycycline also inhibits cell proliferation, migration and matrix metalloproteinase 2 (MMP-2) activity *in vivo* model (Male Sprague-Dawley rats) treated with 30-mg/kg/day doxycycline after arterial injury (125), suggesting that if this drug were to be used for cancer therapy, its toxic side effects might be negligible (119, 126, 127). Mechanistically, doxycycline inhibits matrix-metalloproteinases (MMPs) and the formation of the tumor-sphere in cellular models of OC like SKOV3, ES2 and Tov21G (119). Doxycycline from 50 μM to 500 μM did not affect the viability of normal fibroblasts (hTERT-BJ1) and MCF7 cells (119). Doxycycline treatment reduced tumor growth by 80% in pancreatic tumor xenografts of PANC-1 cells (128). The antibiotic also reduced bone and bone-associated soft-tissue tumor mass by >60% and ~80%, respectively, in a xenograft model of breast cancer bone metastasis that involved MDA-MB-231 cells (129). The anti-cancer activity of doxycycline was attributed to the inhibition of MMPs rather than the targeting of mitochondrial biogenesis (119). Doxycycline exhibited a marked suppression of both invasive and migratory behaviors in human oral squamous cell carcinoma (SCC-15 cells) *in vitro*, with inhibition levels exceeding 75% at a concentration of 10 μg/ml. Additionally, daily administration of doxycycline at a dosage of 3 mg/mice effectively impeded tumor progression in SCC-15 xenografted nude mice, resulting in an 85.6% inhibition rate. Following doxycycline treatment, *MMP-9* mRNA levels in fresh tumor tissue notably decreased compared to the control group treated with normal saline (P < 0.01), while *MMP-2* mRNA levels remained unchanged (130).

Glycylcyclines and tetracyclines impair protein synthesis in bacteria (131, 132). These molecules bind to the bacterial 30S ribosomal unit, inhibiting the binding of aminoacyl-tRNA to the ribosomal A-site. Thus, glycylcyclines could be used to inhibit mitochondrial biogenesis in a similar manner to the one discussed above (119). Pyrvinium pamoate is an anti-helminthic drug which inhibits OXPHOS under normoxic and hypoxic environments and also prevents the formation of the tumor-sphere (119). Finally, tigecycline was shown to also inhibit the formation of a mammo-sphere in SKOV3, ES2 and Tov21G OC cell lines (119).

Monensin is primarily used as a veterinary antibiotic and feed additive for livestock, especially in the prevention and control of coccidiosis. The use of Monensin in human medicine is not authorized, and it is not prescribed for human consumption. However, experiments in SKOV-3, A2780, OVCAR-3 and CAOV-3 cells yielded promising results as a potential repurposed drug against OC. Monensin was shown to regulate the expression molecules linked to the epithelial-mesenchymal transition (EMT) and the mitogen-activated protein kinase (MEK)-extracellular signal-regulated kinase (ERK) pathway (61). This drug inhibited the proliferation of A2780, OVCAR3 and CAOV-3 cell lines from 0.2 µM (low inhibitory effect) until 5 µM (complete inhibitory effect) and impaired the invasive properties of SKOV-3 cells. Furthermore, *in vivo* experiments where SKOV-3 cells were injected into nude mice, followed by monensin administration (0, 8, and 16 mg/kg), resulted in reduced tumor masses in monensein-treated animals, compared to control groups (61). Mechanistically, monensin stimulated the SUMOylation of MEK1, impaired the growth, migration, and invasive capabilities of the A2780, OVCAR3, CAOV-3 and SKOV-3 OC cell lines and in the *in vivo* murine ovarian cancer xenograft model. Thus, monensin holds promise for OC treatment by augmenting MEK1 SUMOylation by suppressing the MEK-ERK signaling pathway (61). To investigate the potential SUMOylation of MEK in OC cell lines, MEK1 and SUMO1 were co-expressed in the non-tumorous cell line HEK293. Immunoprecipitation and western blot analyses of MEK1 revealed that monensin augmented the SUMOylation of MEK1, in a dose- and time-dependent manner (61). However, this drug still needs to be evaluated for approval and usage in human patients.

Bithionol is an anthelmintic drug, historically used for the treatment of intestinal worm infections (133). Bithionol is believed to interfere with the energy metabolism of the parasites, leading to their death. Bithionol has also been used as an antibacterial and antifungal agent in some topical formulations (134, 135). However, due to potential side effects and the availability of other effective treatments, its use in medical practice has been limited. Bithonol causes cell death via caspases-3/7-mediated apoptosis, arrest cell cycle progression, promote the production of Reactive Oxygen Species (ROS) and inhibits Autotaxin (ATX) (136). Autotaxin is an enzyme involved in the production of the signaling molecule lysophosphatidic acid (LPA). ATX and LPA have been implicated in cancer progression, including tumor growth, invasion, and metastasis (137–146). ATX is often overexpressed in several types of cancer, and elevated levels of LPA have been associated with promoting cancer cell survival, migration, and angiogenesis (147–149). In human OC biopsies LPA_2_ and LPA_3_ are highly expressed in comparison with normal ovaries or low malignancy tumors. Furthermore, there is a significant correlation between the expression ratios of LPA_2-3_ and VEGF in patients with cancer (150). Thus, research has been focused to understand ways to inhibit ATX or block the LPA pathways to impede cancer progression. Since ATX is associated with an increase in invasiveness and aggressiveness of tumor cells, and with the grade of tumor development, the inhibition of ATX by bithionol might have an important repercussion in OC treatment (151). In addition, bithionol has been shown to also induce DNA fragmentation, loss of mitochondrial potential and overexpression and activation of apoptotic biomarkers, such as cleaved PARP and caspase-7 (136, 151).

Itraconazole, an anti-fungal drug, has an anti-angiogenic activity and inhibits the Hedgehog pathway inducing autophagic growth arrest (152–155). This drug has been proposed for the treatment of several cancers such as leukemia, ovarian, breast and pancreatic (156). Out of 55 patients with refractory OC, 19 individuals received a combination of itraconazole and chemotherapy. The median progression-free survival (PFS) was 103 days for those receiving chemotherapy (platinum and taxane administration) with itraconazole, compared to 53 days for those without itraconazole (p=0.014). Similarly, the median overall survival was 642 days and 139 days for patients with and without itraconazole, respectively (p=0.006). A proportional hazards regression model (Cox) was employed for multivariate analysis of progression-free survival (PFS) and overall survival (defined as the duration from the commencement of chemotherapy after becoming refractory to death by any cause) following itraconazole exposure alongside chemotherapy. The analysis was adjusted for factors including age, race, Eastern Cooperative Oncology Group (ECOG) performance status (PS), carcinoma histology, number of prior regimens, and platinum sensitivity status. The study demonstrated that the hazard ratio for PFS was 0.24 (p=0.002), and for overall survival, it was 0.27 (p=0.006) in the group receiving itraconazole therapy (157). This data strongly suggested that combining classic chemotherapy with itraconazole may improve the median overall survival rate due to a potential synergistic effect of itraconazole in the treatment of refractory OC (157).

The antiparasitic drug ivermectin, which binds to the glutamic acid operative chloride ion channel (GluCls) (158, 159) has been repurposed for OC treatment (160–162). Ivermectin arrests cell cycle at G0-G1 phase by increasing the synthesis of p21, reducing proliferating cell nuclear antigen (PCNA), cyclin E, and cyclin D protein levels in breast cancer cell lines (MCF-7, MDA-MB-231 and MDA-MB-468) (163). Ivermectin also reduces viability and colony-forming ability in cancer stem-like malignant populations in the SKOV-3 cellular model (163). Furthermore, ivermectin inactivates the P21 (RAC1) Activated Kinase 1 (PAK1), resulting in the inhibition of the phosphorylation of kinase Raf1 (RAF-1) in TYK-nu and RMUG-S OC cells (160). The proliferation rate of TYK-nu, KOC7c, SKOV3, and RMUG-S cell lines was also diminished (160). Ivermectin targets the yes-associated protein 1 (YAP1) (164), which promotes tumorigenesis in breast and liver cancers (165, 166), suggesting a potential application in the treatment of OC, where YAP1 is considered a prognostic marker of the disease (167, 168). Mechanistically, ivermectin blocks the activity of Karyopherin Subunit β1 (KPNB1) in the OC model SKOV3 and OVCAR3 cells, impairing proliferation by targeting several signal pathways, related to cell cycle progression and inducing apoptosis (169, 170). When combined with paclitaxel, these compounds present a synergistic anti-tumor action (171).

Mebendazole is another antiparasitic drug with anti-cancer activity. In several cultured cellular models of OC (MESOV, ES2, A2780, SKOV3 null p53, SKOV3 R248W p53, and SKOV3 R273H p53), mebendazole hindered cell proliferation and activated apoptosis via p53-independent induction of p21 and tubule depolymerization (172). The premise behind exploring these drugs as potential cancer treatments lies in their ability to destabilize microtubules (173–175). Importantly, mebendazole also inhibited cell proliferation and migration in the cisplatin-resistant human OC cell lines OVCAR8CR and OVCAR8 and further induced apoptosis in OVCAR8CR and SKOV3CR cells (176). Mechanistically, this drug modulates essential signaling pathways, such as MYC (Basic Helix-Loop-Helix protein transcription factor)/MAX (MYC Associated Factor X), ELK (ETS (E-twenty six) transcription factor)/SRF (Serum Response Factor), E2F (transcription factor)/DP1 (differentiation regulated transcription factor protein), and nuclear factor kappa B (NF-κB) (176). In addition, in a xenograft murine model of athymic nude mice injected with SKOV3CR cells, mebendazole acted cooperatively with cisplatin to inhibit proliferation, promote apoptosis, and decrease ovarian tumor growth (176). These findings support the possible application of mebendazole in the treatment and maintenance of OC (172), which in combination with cisplatin holds promise for treating chemoresistant OC cases (176).

Peroxisome proliferator-activated receptors (PPARs) have a crucial role in ovarian physiology by regulating the expression and activation of proteases (177–179). PPARγ is regulated by the luteinizing hormone and is highly expressed during ovulation (180). Furthermore, PPARγ(+/−) mice exhibited an approximately 3-fold rise in mammary adenocarcinomas (P<0.05), a more than 3-fold increase in ovarian granulosa cell carcinomas (P<0.05), a greater than 3-fold increase in malignant tumors (P<0.02), and a 4.6-fold elevation in metastatic incidence (181). In mice, PPARγ(+/−) has an increased susceptibility to ovarian carcinogenesis generated by dimethyl benzanthracene (DMBA, 7,12-dimethylbenz[α]anthracene) (181). In a murine model of PPARγ heterozygous female knockout (Pparγ^+/−^) and congenic wild-type littermate controls (Pparγ^+/+^), treated with the carcinogen DMBA, at the 25^th^ week from the initiation of the study, KO mice exhibited significantly higher skin papilloma multiplicity (0.87 papillomas/mouse) compared to controls (0.52 papillomas/mouse; P<0.05) (182). By the end of the observation period, ∼41% (18 out of 44) of controls (*Pparγ* ^+/+^) and ∼61% (24 out of 39) of knockout (*Pparγ^+/−^*) mice died or had to be killed due to morbidity resulting from tumor progression (181). Tumors in Pparγ^+/−^ mice were found to be in a more advanced state compared to wild-type controls. Although the total ovarian tumor multiplicity did not differ between the two genotypes, Pparγ^+/−^ mice displayed a significantly higher multiplicity of malignant tumors per mouse compared to wild-type controls when tumors were categorized as benign or malignant. In particular, among the total ovarian tumors, there were 3 carcinomas out of 12 in wild-type mice and 10 carcinomas out of 13 in Pparγ^+/−^ mice, reaching a significance level of P < 0.02 (181). The increased susceptibility of Pparγ^+/−^ mice to DMBA-induced carcinogenesis implies that PPARγ may function as a tumor modifier. Consequently, PPARγ-specific ligands could potentially play a beneficial role in chemo preventing ovarian carcinogenesis (181.

Activating ligands of PPARγ, such as ciglitazone, pioglitazone, and *t*-butyl [1,1-bis(3′-indolyl)-1-(*p-t*-butyl)methane (DIM-C-pPhtBu) have been proposed to inhibit OC by impairing proliferation and tumor development and also by triggering apoptosis (183–186). Specifically, ciglitazone inhibits cell proliferation by blocking cell cycle progression and promoting apoptosis (183, 184). In addition, ciglitazone enhanced PAR1-triggered prostaglandin E2 (PGE2) production and cyclooxygenase 2 (COX-2) expression in the normal rat gastric epithelial cell line (RGM1) (187).

Treatment with ciglitazone reduces *Cox-2* mRNA expression and PGE2 production, while also decreasing COX-2 promoter activity. Additionally, it upregulates PPRE (putative PPAR response element) promoter activity in human non-small-cell lung cancer cells (A427 and A549) (188). Ciglitazone decreases expression levels of glucose transporter-1 (GLUT-1), inhibits glucose uptake, and increases tumor cell apoptosis in A2780 and OVCAR3 OC cells (189). Additionally, it reduces expressions of specific protein 1 (Sp-1) and β-catenin while increasing phosphorylation levels of adenosine monophosphate (AMP)-activated protein kinase and enhancing chromatin condensation and fragmentations (189). In an *in vivo* model utilizing eight-week-old female NOD-scid IL2R γ null (NSG) mice injected with A2780 OC cells, ciglitazone significantly decreases OC mass transplanted onto the back of the mice. GLUT-1 expression is increased in high-grade serous ovarian carcinoma, with expression levels proportional to cancer stage severity (189). Mechanistically, DIM-C-pPhtBu induces PPARγ-dependent p21 and reduces PPARγ-independent cyclin D1, resulting in cell cycle arrest, inhibition of cell proliferation, and apoptosis induction (185).

Clofibric acid, commonly used for the treatment of hyperlipidemia, was shown to reduce the growth of OVCAR-3 tumors transplanted subcutaneously and notably prolonged the survival of cancerous peritonitis mouse model with malignant ascites originating from DISS cells compared to the control group (190). Moreover, clofibric acid exhibited dose-dependent suppression of cell proliferation in OVCAR-3 cells. In both implanted OVCAR-3 tumors and cultured OVCAR-3 cells, clofibric acid treatment induced the expression of carbonyl reductase (CR), which promotes the conversion of PGE2 to PGF2α (prostaglandin F2α) (190). Clofibric acid treatment also reduced the levels of PGE2 and VEGF in OVCAR-3 tumors and DISS-derived ascites. Solid OVCAR-3 tumors treated with clofibric acid exhibited reduced microvessel density and increased apoptosis (190).

Disulfiram, a medication used to treat alcohol dependence, has been studied for its potential anticancer effects in OC. Research suggests that disulfiram may inhibit cancer cell growth and metastasis in copper (Cu)-dependent manner due to its ability to bind this ion (191, 192). Approximately 60% of breast cancer patients have elevated levels of Cu in serum (average 3.25 μg/mL) compared with healthy individuals (average 2 μg/mL) (193). The disulfiram-Cu complex was shown to be is a potent inhibitor of proteasomal activity and to trigger apoptosis in the cultured breast cancer cell lines MDA-MB-231 and MCF10DCIS.com, with no effect in non-tumorigenic immortalized MCF-10A cells (192). In mice with MDA-MB-231 tumor xenografts, disulfiram notably suppressed tumor growth by 74%. This effect was attributed to apoptosis induction and proteasome inhibition, which rendered accumulation of ubiquitinated proteins and the natural proteasome substrates p27 and Bax (BCL2 associated X, apoptosis regulator) (192). Disulfiram-Cu complex increases intracellular Cu concentration both *in vitro* and *in vivo*, bypassing the requirement for Cu-membrane transporters, such as Ctr1, suggesting that the classical transporter Ctr1 may not play a significant role in disulfiram-mediated Cu accumulation (194, 195). This complex antagonized NFκB signaling, suppressed aldehyde dehydrogenase activity and antioxidant levels, thereby inducing apoptosis mediated by oxidative stress in the inflammatory breast cancer model SUM149, rSUM149 cells (194, 195).

In a murine model, the disulfiram-Cu complex markedly suppressed tumor growth without notable toxicity, inducing apoptosis exclusively in tumor cells. This underscores that inflammatory breast cancer tumors are highly redox-adapted, potentially conferring resistance to ROS-inducing therapies (194, 195). Hence, the redox modulation capabilities of disulfiram represents a promising avenue for treating tumors enhancing the efficacy of traditional therapies (192). As such, in cultured OC models, disulfiram promoted oxidative stress through an Cu-dependent mechanism, resulting in death of OVCAR-3, SKOV-3, OVMZ-30, OVMZ-31, OVMZ-37, and OVMZ-38 cells (196). It has been reported that the ditiocarb-Cu complex, a metabolite of disulfiram, is responsible for the anti-cancer effects. Additionally, functional, and biophysical analyses identified NPL4 (nuclear protein localization protein 4 homolog) as the molecular target underlying the tumor-suppressing effects of disulfiram (191). NPL4 acts as an adaptor of p97, also known as ATPase valosin-containing protein (VCP) segregase, which is crucial for protein turnover in various regulatory and stress-response pathways within cells (191).

Disulfiram, combined with Cu, enhances cisplatin-induced apoptosis in IGROV1, SKOV3, and SKOV3IP1 cells, sensitizing cancer cells to cisplatin treatment and decreasing cell viability by 50-80%% (197). This combination targets acetaldehyde dehydrogenase (ALDH)+ cells, favoring cisplatin sensitivity in H460/CisR, H1299/CisR, and SKMES-1/CisR cells (198). Additionally, disulfiram and Cu supplementation reduces NF-κB activity and sensitizes H630WT and HCT116WT cell lines to gemcitabine (199). Disulfiram reverses doxorubicin (DOX) resistance by increasing c-Jun NH2-terminal kinase (JNK) expression and phosphorylation in HL60 cells (200). Effective cell death in OVCAR3 and SKOV3 cells is mediated by disulfiram, promoting an oxidative intracellular environment and causing irreversible cell damage associated with the expression of heat shock proteins HSP32, HSP40, and HSP70 (196). Furthermore, the combination of disulfiram and Cu, induces disulfide bond-mediated dimerization of HSP27, resulting in its inactivation and rapid detachment of OVCAR-3 cells, an effect not detected with disulfiram alone (196). Combinatory treatment of disulfiram with auranofin, an anti-rheumatic drug, enhances cytotoxic effects in OVCAR3 cells (188).

Fluphenazine is an antipsychotic drug that exerts its effects on the postsynaptic dopaminergic D1 and D2 receptors by inhibiting the release of dopamine. In OC OVCAR-3 cells, fluphenazine plays an essential role in the phosphorylation of AKT dependent on epidermal growth factor (EGF) (201, 202). Moreover, fluphenazine targets pyruvate dehydrogenase kinase 1 (PDK1), which is part of the PDK1/Akt pathway mediating cell survival, proliferation and tumorigenesis (201). Thus, the inhibition of PDK1/Akt kinase pathway suppressed the EFG-dependent proliferation phenotype and survival of cancer OVCAR-3 cells by inducing apoptosis (201). The proposed mechanism of action for fluphenazine is related to an enhancement of genomic DNA-oligonucleosomal cleavage, and to the activity of the caspase substrate polyadenosine diphosphatase ribose. These pathways trigger caspase-dependent apoptotic cell death (201).

Metformin, a frequently prescribed medication for type 2 diabetes mellitus, reduces proliferation of SKOV3ip1, OVCAR-5, HeyA8 and K-ras/PTEN cells. The K-ras/PTEN mouse OvCa cell line was established from ovarian tumors generated using a genetic mouse model (203, 204)). Mechanistically, metformin causes cell cycle arrest in G0/G1 phase by decreasing the expression of cyclin-dependent kinase 4 (CDK4) and Cyclin D1, with no evidence of triggering apoptosis (204). Metformin treatment results in reduced number and mass of ovarian tumors. For instance, female athymic nude mice pretreated with metformin (250 mg/kg/d) exhibited significantly fewer ovarian tumor implants compared to controls (mean number of tumors: placebo, 116; metformin, 47; P<.005) (204). In SKOV3ip1 xenograft mice, treatment with metformin in combination with paclitaxel resulted in a 60% reduction in tumor weight compared to controls (P=.02) (204). This combination demonstrated a stronger effect than each compound tested separately (204). Metabolically, metformin modifies adenosine monophosphate-activated protein kinase (AMPK) activity, lipid synthesis, and glycolysis. Notably, metformin induces apoptosis in OC cell lines in an AMPK-dependent manner (205–207).

Furthermore, a study of OC patients where a cohort of individuals was treated with metformin resulted in an increased survival rate (67%) of individuals treated with metformin compared to the non-treated group (47%) (208). Patients who consumed this drug exhibited a markedly enhanced 5-year survival rate (51%) compared to those who did not use metformin (8%) or those without diabetes (23%) (209). In combined metformin/cisplatin treatment, increasing metformin concentrations led to a notable reduction in the half-maximal inhibitory concentration of cisplatin (209). Consequently, research in ovarian cancer patients, alongside *in vivo* and *in vitro* models, highlights the inhibitory effect of metformin on tumor growth, its ability to enhance chemotherapy sensitivity, and its potential to prolong the life expectancy of affected individuals.

Naftopidil is an α1-adrenergic receptor antagonist that is primarily used for the treatment of benign prostatic hyperplasia (BPH), a condition characterized by an enlarged prostate gland in men (210–212). By blocking the α-1 adrenergic receptors in the prostate, naftopidil helps to relax the smooth muscles in the prostate and bladder neck, relieving symptoms of BPH and improving urine flow (213). In studies using cellular models of OC, naftopidil inhibited proliferation without eliciting apoptosis, leading to a cytostatic effect observed in SKOV-3 and IGROV1-R10 cell lines (214). Furthermore, this medication enhances the production of proapoptotic BH3-only proteins, namely Bim (BCL2-like 11, member of the Bcl-2 family that promotes apoptosis), Noxa (phorbol-12-myristate-13-acetate-induced protein 1), and Puma (BCL2 binding component 3). Two different mechanisms have been identified for naftopidil in OC-cultured models. For instance, in SKOV3 cells, an ER stress-induced response by the activating transcription factor 4 (ATF4), which is responsible for the phenotype, while in the IGROV1-R10 cell line, the JNK pathway is the leading pathway (214). Considering the mechanisms by which naftopidil induces the expression of Puma by the JNK/c-Jun pathway, resulting in a new alternative to OC management (214).

Nelfinavir is a protease inhibitor primarily used in the treatment of HIV (human immunodeficiency virus). Experiments in HGSOC cells showed that treatment with this drug reduces the cell number, clonogenic survival and viability (215). Additionally, nelfinavir favors a pro-apoptotic environment characterized by elevated levels of phospho-eIF2α (Eukaryotic Translation Initiation Factor 2A), DNA Damage Inducible Transcript 3 (DDIT3, also known as CHOP), and ATF4, as well as an increased ratio of Bax/Bcl-2 and cleaving of the executor caspase 7 (215). Nelfinavir triggered a dose-dependent reduction in the HGSOC cell number and viability and a parallel increase in hypo-diploid DNA content, independently of platinum sensitivity (215). DNA damage induced by nelfinavir was detected by the phosphorylation of the histone marker, H2AX (H2A.X variant histone) in PEO1 and PEO4 cell lines, in a process linked to reduced proliferation and survival mediated by the ERK and AKT pathways (215). In the PEO1 and PEO4 cellular models, a synergistic effect of nelfinavir with the protease inhibitor, bortezomib, enhanced the ability to induce short-term cell cycle arrest and long-term toxicity (215). So far, bortezomib has been used in the treatment of multiple myeloma and mantle cell lymphoma, but similarly to nelfinavir possess the potential as a treatment against OC.

Ritonavir is another protease inhibitor, largely used in the treatment of HIV, in combination with other antiretroviral medications to slow the progression of the disease. Ritonavir inhibits the activity of the HIV protease enzyme, which is necessary for the virus to replicate and produce new infectious viral particles (216). By inhibiting protease, Ritonavir helps reduce the viral load in the body. Additionally, ritonavir is often used as a “booster” medication, as it increases the levels of other protease inhibitors in the blood, enhancing their effectiveness. This boosting effect allows for lower doses of other protease inhibitors when used in combination with nucleoside analog reverse transcriptase inhibitors (NRTI), resulting in highly effective antiretroviral therapy. In the context of OC repurposing, ritonavir was shown to prevent cell cycle progression in MDH-2774 and SKOV-3 cultured models (217). Furthermore, in MDH-2774 and SKOV-3 cell lines, this drug promoted apoptosis and cell cycle arrest in G1 phase by depleting the phosphorylation of retinoblastoma (RB), and by reducing the expression of G1 cyclin and cyclin-dependent kinase (217). In MDAH 2774 and SKOV-3 cells, ritonavir also increased levels of phosphorylated AKT, thus inhibiting the PI3K-AKT pathway, which resulted in an antitumor effect that led to apoptosis (217–219). In xenograft models, nude mice injected with human ovarian adenocarcinoma A2780 cells and treated with ritonavir exhibited reduced tumor burden compared to untreated controls. Additionally, ritonavir-treated mice showed larger areas of necrosis and increased activated caspase-3 staining, indicating induction of apoptosis in the tumor cells (219).

Non-steroidal anti-inflammatory drugs (NSAIDs) are a class of medications commonly used to reduce inflammation, relieve pain, and lower fever. NSAIDs inhibit the production of prostaglandins by blocking cyclooxygenases (COX), which play a role in inflammation and pain (220). Thus, NSAIDs have anti-inflammatory and analgesic (pain-relieving) properties, often used to manage conditions characterized by inflammation, such as arthritis, osteoarthritis, rheumatoid arthritis, and to alleviate systemic pain in cases of menstrual cramps, headaches, muscle aches, and minor injuries (221–223).

Examples of NSAIDs include ibuprofen (e.g., Advil, Motrin), naproxen (e.g., Aleve), aspirin, diclofenac, and meloxicam. Among these, aspirin has been investigated for its potential to reduce the risk of ovarian cancer development and progression. Aspirin inhibits NF-κB, COX, and the PI3K/mTOR signaling pathway, concurrently activating AMPK (224). Some studies suggest that regular aspirin use may be associated with a reduced risk of OC incidence and mortality (225). Aspirin was shown to inhibit the proliferation of OCT2 and OVCAR-3 cells and to reduce PPARδ function by inhibiting ERK1/2 (226). Therefore, NSAIDs show promise as therapeutic treatments against OC; however, dosage seems to be a key feature requiring further investigation (158).

Cancer chemotherapeutic treatment often results in a significant upregulation of transmembrane efflux pumps, which contribute to the development of multiple drug resistance, a major impediment in effective cancer treatment. Highly resistant tumors might be eradicated using chemosensitizers that block the efflux of the drug and increase the entry of the drug into the cell (227). In this regard, sertraline is a selective serotonin reuptake inhibitor, commonly prescribed for the treatment of various mental health conditions, particularly depression, anxiety disorders, obsessive-compulsive disorder, and panic disorder (228). Sertraline increases the levels of serotonin, a neurotransmitter in the brain, which is believed to have a positive impact on mood and emotional well-being (229). For instance, P-glycoprotein (P-gp) is a transmembrane efflux pump that actively transports and eliminates drugs and other chemical compounds from cells. This protective function prevents the buildup of potentially harmful substances within cells, negatively impacting the therapeutic effectiveness of the drugs. Thus, P-gp is linked to multidrug resistance observed in cancer cells that develop resistance to multiple chemotherapeutic drugs (230). The significant implications for pharmacokinetics, where P-gp influences the absorption, distribution, and elimination of drugs, lead to altered bioavailability and distribution patterns for drugs that are substrates for P-gp (231). Certain drugs can either inhibit or induce P-gp activity, affecting the cellular concentrations of various substrate drugs. Ongoing research focuses on P-gp in drug development to enhance drug efficacy and address multidrug resistance, with efforts directed at designing drugs that can bypass or inhibit P-gp when necessary. This knowledge is essential for healthcare professionals and researchers navigating drug interactions and optimizing therapeutic outcomes. P-gp pumps are expressed and functional in the chemoresistant ovarian adenocarcinoma cell line OVCAR-8 and in the derived drug-resistant models (human ovarian adenocarcinoma cell line NCI/ADR-Res (NAR) cells) (227). Among these, sertraline has been shown to enhance the cytotoxicity of DOX and reduce DOX efflux in NAR cells (227). Studies conducted in human ovarian adenocarcinoma xenograft models demonstrated that combining sertraline with DOXIL^®^ (pegylated liposomal DOX) effectively reverses multiple drug resistance (MDR). Sertraline acts as a chemosensitizer by blocking extrusion pumps, thereby allowing the drug delivered via the nanomedicine to accumulate inside the cell (227). Hence, the combined therapy of nanomedicine with chemosensitizers like sertraline is poised to amplify therapeutic responses in highly resistant tumors. This approach increases drug influx through nanomedicine while reducing drug efflux by employing a chemosensitizer (227). Moreover, findings from a xenograft murine model revealed that combining sertraline with DOX significantly enhances cytotoxicity, delaying tumor growth and improving survival rates by 1.5-fold (227). This combined treatment holds promise in mitigating multiple drug resistance phenotypes attributed to P-gp pumps, such as Multidrug Resistance 1 (MDR1, also known as ABCB1), which are ATP-dependent efflux pumps of the ABC protein superfamily (227, 232).

Thalidomide was initially marketed as a sedative-hypnotic drug with anti-emetic activity against morning sickness of early pregnancy, but was withdrawn from the market in the early sixties as it was found to cause severe fetal malformations (233–235). It is a medication with immunomodulatory and antiangiogenic properties that has been investigated for its potential to inhibit tumor growth and angiogenesis in ovarian cancer. Studies suggest that thalidomide may exert anticancer effects by modulating immune responses and disrupting tumor microenvironment interactions (10). Thalidomide inhibits TNF-α production in lipopolysaccharide-stimulated monocytes (236). Thalidomide decreased the capacity of SKOV-3 cells and primary epithelial ovarian carcinoma cells to secrete TNF-α, but this drug did not significantly affect the proliferation and growth of SKOV-3 cells (237). Thalidomide notably decreased the capacity of SKOV-3 cells to secrete MMP-9 and MMP-2, yet it did not have the same effect on primary epithelial ovarian carcinoma cells. However, thalidomide did not affect the secretion of IL-6 in either SKOV-3 cells or primary epithelial ovarian carcinoma cells (237). Thalidomide inhibits the processing of the *TNF-α* and the angiogenic factor *VEGF* transcripts (238). Sixty-six patients, comprising 37 women and 29 men, with advanced cancer (19 ovarian, 18 renal, 17 melanoma, 12 breast cancer) received daily treatments ofthalidomide at a dose of 100 mg. Out of the 18 patients with renal cancer, three showed partial responses, and an additional three patients experienced disease stabilization for up to 6 months. Although no conclusive responses were observed in patients, there was an improvement in the sleep quality (P<0.05) and preserved appetite (P<0.05) in these individuals (239). Women (138) diagnosed with biochemical-recurrent epithelial OC, primary peritoneal cancer, or fallopian tube carcinoma were eligible for a randomized phase III trial of tamoxifen versus thalidomide (240). Results suggested that thalidomide treatment was associated with a similar risk of progression (HR=1.31, 95% confidence interval [CI]=0.93–1.85), an increased risk of death (HR=1.76, 95% CI=1.16–2.68) and more grades 3 and 4 toxicities (55% versus 3%) in comparison with tamoxifen treatment (240). Therefore, thalidomide was not more effective than tamoxifen in delaying recurrence or death but was more toxic (240).

## Repurposed kinase inhibitors

Several kinase inhibitors, originally developed for different pathologies, have been investigated for their potential to target specific signaling pathways implicated in OC. Examples include dasatinib, a Src kinase inhibitor, and imatinib, a BCR-ABL tyrosine kinase inhibitor, which have shown promise in preclinical studies of ovarian cancer (241).

Dasatinib is an inhibitor of Src/Abl family kinases used for the treatment of Philadelphia chromosome-positive acute lymphoblastic leukemia or chronic myeloid leukemia (242). Dasatinib inhibited cell growth by partially inducing apoptosis with a significant effect in autophagy activation in the SKOV3 and Hey cell lines (243). Dasatinib reduced the phosphorylation of AKT, mTOR, p70S6K, and S6 kinase expression and reduced Bcl-2 expression and activity. Dasatinib induces autophagy in Hey and SKOV3 cells that partially depends on beclin 1, AKT and Bcl-2. Overexpression of Bcl-2 partially prevented dasatinib-induced autophagy. In a Hey xenograft model, dasatinib inhibited tumor growth and induced both autophagy and apoptosis (243). Elevated levels of p-Src (phosphorylated Src family tyrosine kinases) protein expression were detected in A2780, HO8910, OVCAR3, CAOV3, and COC1 cell lines compared to healthy cells. This observation suggests activation of the Src signaling pathway (244). Combining dasatinib and paclitaxel significantly inhibited proliferation and boosted apoptosis in A2780 and HO8910 cells compared to controls. This combination showed tumor growth inhibitory rates of 76.7% and 58.5% in A2780 and HO8910 cell lines, respectively, outperforming paclitaxel treatment alone (244). In A2780 and HO8910 xenografts models, dasatinib treatment inhibited tumor growth by 43.2% and 34.0%, respectively (244). Paclitaxel treatment increased Src activation in Hey OC cells, inducing the expression of EpCAM (epithelial cell adhesion molecule) marker expression in Hey cells, while upregulated the expression of SSEA-4 (stage-specific embryonic antigen-4) and CD133 (prominin 1) markers (245). In this sense, dasatinib combined with paclitaxel significantly suppressed p-Src in Hey cells and xenografts but had no effect on the expression of these markers (245). However, this combination did not enhance the proliferative, tumorigenic, and vasculogenic of paclitaxel alone in HEY cell-induced ovarian tumors (245). Importantly, administration of dasatinib and paclitaxel in murine models reduced the invasion of cancer cells into the pancreas and liver, major organs affected by ovarian tumor metastasis. Thus, the evidence points to a significant potential of dasatinib in targeting intra-peritoneal dissemination of OC (245).

Imatinib inhibits the proliferation of several OC cell lines (C272-hTert/E7, C889/hTert, CSOC848, CSOC908, and CSOC918) that expressed elevated levels of PDGFRα (platelet-derived growth factor receptor α) (246). SKOV3 and CAOV3 cells do not express PDGFRα are insensitive to the effects of imatinib, suggesting that the inhibition of cell proliferation by imatinib is in a PDGFRα-specific manner. Imatinib induces antiproliferative effects by arresting cell progression at G0-G1 and impeding advancement through the S phase. Additionally, at a concentration of 1 μm, Imatinib inhibits both PDGFRα and Akt phosphorylation (246). However administration of imatinib to patients with epithelial OC, had minimal effect as a single treatment (247). A phase II trial of imatinib administered to patients with platinum-resistant OC, showed that imatinib mesylate, when used alone, lacks significant clinical efficacy in c-Kit and/or PDGFR positive, recurrent OC, particularly in heavily pretreated patients (248). Thus, imatinib may be considered as a supplementary drug to be used in combination with other treatments.

Hormonal therapy is an emerging treatment that utilizes hormones or hormone-blocking agents to interfere with the growth and progression of OC cells. While hormonal therapy is not a standard treatment for most OC, it may be considered in specific cases where the cancer cells express hormone receptors, such as ER and PR. This strategy can be particularly useful for endometrioid OC and some ovarian stromal tumors that may express these hormone receptors. Among the drugs utilized in hormonal therapy is tamoxifen, a Selective ER Modulator (SERM) commonly used in breast cancer treatment and has been investigated in some cases of OC with hormone receptor expression (249, 250). Aromatase inhibitors, such as letrozole and anastrozole, which prevent the synthesis of estrogen, are mainly used in the treatment of breast cancer and oftentimes as fertility treatments, are sometimes used in ER+ OC cases (251–255). In a phase II trial involving 50 patients with relapsed ovarian cancer, the antitumor activity of letrozole was assessed using Union International Contre Cancer (UICC) and CA125 (cancer antigen 125) marker criteria. Tumors categorized as stable disease by UICC criteria showed significantly higher ER (P=0.027) and PR (P=0.0066) values compared to those categorized as progressive disease (251). The combined presence of these receptors strongly correlated with stable disease (P<0.0001). Similarly, according to CA125 criteria, tumors with higher ER (P=0.013), lower erbB2 (P=0.026), and higher epidermal growth factor receptor (P=0.009) levels were associated with CA125 stable/responsive disease compared to progressive disease (251). In another phase II trial, letrozole was administered at a daily dosage of 2.5 mg until either clinical or marker evidence indicated disease progression. This trial focused on ER-positive OC patients with rising CA125 levels, indicative of progression according to Rustin’s criteria (252). Among the 42 patients assessed for CA125 response, 7 (17%) showed a response, defined as a decrease of more than 50%, while 11 (26%) patients did not experience progression, indicated by a doubling of CA125 levels, after 6 months of treatment (252). Of the 33 patients evaluable for radiological response, 3 (9%) had a partial remission, and 14 (42%) had stable disease at 12 weeks (252). Subgroup analysis based on ER status showed CA125 response rates of 0% (immunoscore of 150-199), 12% (immunoscore of 200-249), and 33% (immunoscore of 250-300), with a significant trend observed (P = 0.028, χ2 for trend). Additionally, expression levels of HER2, insulin-like growth factor binding protein 5 (IGFBP5), trefoil factor 1 (TFF1), and vimentin correlated with changes in CA125 levels during treatment (252). Finally, a 2.5 mg daily oral dose of letrozole was administrated to thirty-three women with recurrent ER+ epithelial ovary or peritoneum carcinoma enrolled in a phase II trial (253). Among these patients, 26% of the individuals diagnosed with ER^-+^, platinum- and taxane-resistant high-grade ovarian and primary peritoneal cancer who received letrozole treatment experienced a clinical benefit, defined as either stabilization of disease or partial response (3% of patients) (253).

Ormeloxifene is a SERM primarily used as an oral contraceptive and for the treatment of conditions related to the female reproductive system. It inhibits the action of estrogen on the uterus, leading to changes in the cervical mucus chemistry and endometrium. These physiological changes create a challenging environment for the sperm to reach the egg and for a fertilized egg to successfully implant in the uterus. In the context of OC repurposing, *in vitro* experiments showed that ormeloxifene hindered cell proliferation and triggered apoptosis in cisplatin-resistant in the A2780, A2780-CP and SKOV-3 cell lines (256). At the molecular level, ormeloxifene reduced AKT phosphorylation, enhanced p53 phosphorylation, and altered the synthesis and localization patterns of cyclin D1, cyclin E, p27, and CDK2 (256). In xenograft murine models, injecting 50 or 100 µg ormeloxifene once a week for 5 weeks reduced tumorigenesis and metastasis within the peritoneal cavity (256). Within 2 weeks of A2780-CP cell injection, all mice treated with vehicle displayed a swollen abdomen, indicative of ascites formation, along with significant peritoneal carcinomatosis and numerous solid tumors (256). Conversely, mice treated with 100 µg of ormeloxifene showed no detectable tumors (256). This suggests that ormeloxifene holds promise as a compound for OC treatment. Despite the potential benefits, hormonal therapy is not widely used in OC yet and is only considered when other standard treatments are not effective and in specific cases of OC patients expressing the hormone receptors. It is noteworthy that while hormonal therapy has potential in OC treatment, standard treatments such as surgery and chemotherapy remain the mainstay of ovarian cancer management. As clinical research and trials progress, this treatment option may become an efficient alternative to OC care.

## Model-informed drug repurposing

Model-informed drug repurposing (MIDR) might be used to accelerate the repositioning of drugs (257). MIDR is a rapidly expanding *in silico* approach to drug discovery and development that involves mathematical models, computational tools, and data-driven techniques to identify new therapeutic uses for existing drugs (257). The development of powerful computational methods, such as bioinformatics, systems biology, and quantitative pharmacology modeling, and the combination of these techniques, allow the analysis of large datasets to identify potential connections between drugs and diseases. This is further achieved by consolidating diverse data sources, such as genomic, transcriptomic, proteomic, and clinical records. In addition, network pharmacology, as well as pharmacokinetic and pharmacodynamic modeling, further refine the understanding of complex interactions among drugs, targets, and diseases. Ultimately, these approaches are now being integrated into machine learning algorithms and artificial intelligence pipelines to combine complex datasets and efficiently predict drug-disease relationships. These *in silico* models can also help identify synergistic effects of drug combinations for improved therapeutic outcomes. This powerful experimental approach is now being utilized in the treatment of cancer.

As an example, a recent report that involved a literature search coupled to *in silico* analyses and screening process involving preclinical research, explored the testing of approved compounds for human use in treating OC (258). The combination of these approaches rendered four compounds used regularly in the clinic, metformin, celecoxib, lurbinectedin, and 5-azacytidine, as drugs with significant potential for repurposing in the context of myeloid-derived suppressor cells (MDSC) within the OC tumor microenvironment (258). MDSC suppresses the immune response in OC through several mechanisms; therefore, finding potential drug candidates for repositioning has been a challenging process (258). As example is the emerging evidence for lurbinectedin, a synthetic compound derived from the marine organism *Ecteinascidia turbinate*. lurbinectedin is an alkylating anticancer drug. It targets specific DNA repair mechanisms in cancer cells, leading to DNA damage and cell death (259). Lurbinectedin decreases myeloid-derived suppressor cell (MDSC) percentages *in vitro* in chronic lymphocytic leukemia (CLL) patient-derived studies (260). It induces cell death in a dose and time-dependent manner and reduces the expression of chemokine receptor CCR7 implicated in B-CLL cell migration (260). Notably, malignant B cells from patients with clinical lymph node involvement exhibit higher trans-endothelial cell migration (TEM) in response to CCL21 and CCL19 compared to those without such organomegaly (260). There is a correlation between CCR7 expression, receptor for both CCL21 and CCL19, and clinical lymphadenopathy, and blocking CCR7 suppresses TEM of CLL cells (260). Lurbinectedin has shown promise in treating various solid tumors, including small-cell lung cancer and relapsed OC (261). This drug received accelerated approval from the U.S. FDA for metastatic small-cell lung cancer that has progressed after platinum-based chemotherapy (262). *In vitro* studies revealed significant antitumor effects of lurbinectedin on both chemosensitive and chemoresistant clear cell carcinoma (CCC) of the ovary cells (RMG1, RMG2, KOC7C, and HAC2) (263). Evaluation of mouse CCC cell xenografts confirmed that lurbinectedin effectively suppressed tumor growth. Notably, combining lurbinectedin with SN-38 (7-ethyl-10-hydroxycamptothecin) demonstrated a significant synergistic effect, particularly evident in both cisplatin-resistant and paclitaxel-resistant CCC cell lines. These findings indicate potent antitumor activity of lurbinectedin in both cisplatin-sensitive and cisplatin-resistant OC (263). Furthermore, lurbinectedin is under investigation in clinical trials for potential efficacy in other cancer types, including advanced ovarian, endometrial, and breast cancers, relapsed hematological malignancies like acute myeloid leukemia (AML) and lymphomas, and soft tissue sarcomas including liposarcoma and leiomyosarcoma (261, 264, 265). Combination therapies involving lurbinectedin are being assessed, including with immune checkpoint inhibitors, PARP inhibitors, and other targeted therapies, to explore potential synergistic effects.

Celecoxib, a selective COX-2 inhibitor used to treat pain and inflammation, has been investigated for its potential to inhibit OC growth and metastasis. Evidence suggested that celecoxib may exert anticancer effects in OC cells by inhibiting COX-2-mediated signaling pathways involved in tumor progression (266). In areas of active tumor growth in a murine model for mesothelioma, large numbers of MDSCs co-localize with COX-2 expression (267). Celecoxib effectively reduced PGE2 levels both *in vitro* (mesothelioma AB1 cell line) and *in vivo* (BALB/c mice) (267). Furthermore, celecoxib treatment decreased levels of ROS in immature myeloid subtypes (MO-MDSC, PMN-MDSC, and Gr-1lowSubset 2) from the spleen of tumor-bearing mice and improved cytotoxic T cell function (267). Ten days after injection with a lethal dose of 0.5×10^6^ AB1 tumor cells, the absolute number of MDSCs was significantly lower in mice receiving the celecoxib diet compared with mice receiving the control diet. This difference was more pronounced at day 22 after tumor injection, and mice receiving the celecoxib diet did not exhibit any discernible side effects (267). These findings highlight the potential of celecoxib as an adjunctive therapy in cancer treatment strategies.

Finally, 5-azacytidine is a nucleoside analog that is incorporated into DNA and RNA and inhibits DNA methyltransferase enzymes leading to subsequent DNA hypomethylation. It is primarily used as a demethylating agent in the treatment of certain hematological malignancies, particularly myelodysplastic syndromes, where it can help to restore normal hematopoiesis by reversing aberrant DNA methylation patterns (268–270). The inhibition of methylation via 5-azacytidine increases the formation of invadopodia and enhances the extracellular matrix degradation in SKOV3 and A2780 cells and further promotes cell migration and invasion of SKOV3 cells (271). Moreover, in SKOV3 cells, 5-azacytidine induce the expression of genes and proteins involved in actin-regulating signaling pathways [*PIK3CA* (phosphatidylinositol-4,5-bisphosphate 3-kinase catalytic subunit alpha), *SRC* (SRC proto-oncogene, non-receptor tyrosine kinase), *RhoC* (ras homolog family member C), *RhoA* (ras homolog family member A), *RAC1* (Rac family small GTPase 1), and *AFAP* (actin filament associated protein 1) (271). Furthermore, the 5-azacytidine increased the phosphorylation of AKT and p110 alpha (PI3-kinase isoform), suggesting that the PI3K-AKT pathway is activated in SKOV3 and A2780 cells (271). In mouse xenograft models, 5-azacytidine treatment suppressed tumor growth and increased the occurrence of metastatic nodules, indicating an enhanced metastatic potential due to DNA demethylation (271). Methylation inhibition led to increased transcription of PIK3CA and upregulation of genes associated with the PI3K-AKT signaling pathway (271). This induction likely occurs through epigenetic regulation of *PIK3CA*, as analysis of DNA methylation levels in the *PIK3CA* promoter region indicated decreased methylation of CpG islands in SKOV3 and A2780 cells following 5- azacytidine treatment (271). The impact of trichostatin A (TSA) and 5-azacytidine (5-aza-2′-deoxycytidine), either alone or in conjunction with low-dose cisplatin, was assessed on Hey, SKOV3 and A2780 lines *in vitro* (272). Combined treatment exhibited superior efficacy compared to individual drugs and notably suppressed cell viability, migration, and spheroid formation and growth in Hey, SKOV3 and A2780 cells (272). Sequential administration of cisplatin (1 mg kg^−1^) followed by TSA (0.3 mg kg^−1^) significantly suppressed the tumorigenicity of Hey xenografts by inhibiting the expression of epithelial to mesenchymal transition (EMT) markers (Twist, Snail, Slug, E-cadherin, and N-cadherin), and reducing the pluripotency of ovarian cancer cells (272). Finally, a clinical trial in patients with platinum-resistant OC showed the effect of oral 5-azacytidine in combination with pembrolizumab (NCT02900560). Pembrolizumab is a monoclonal antibody medication used as immune checkpoint inhibitor for immunotherapy of various types of cancer (273). This study helped to establish an optimal dosing schedule for oral azacitidine in combination with pembrolizumab for platinum-resistant/refractory OC patients (274). Additional preclinical studies using 5-azacytidine alone and in combination with a small molecule histone deacetylase (HDAC) inhibitor, entinostat, showed high potential in OC treatment as well (258).

**Figure 5:**
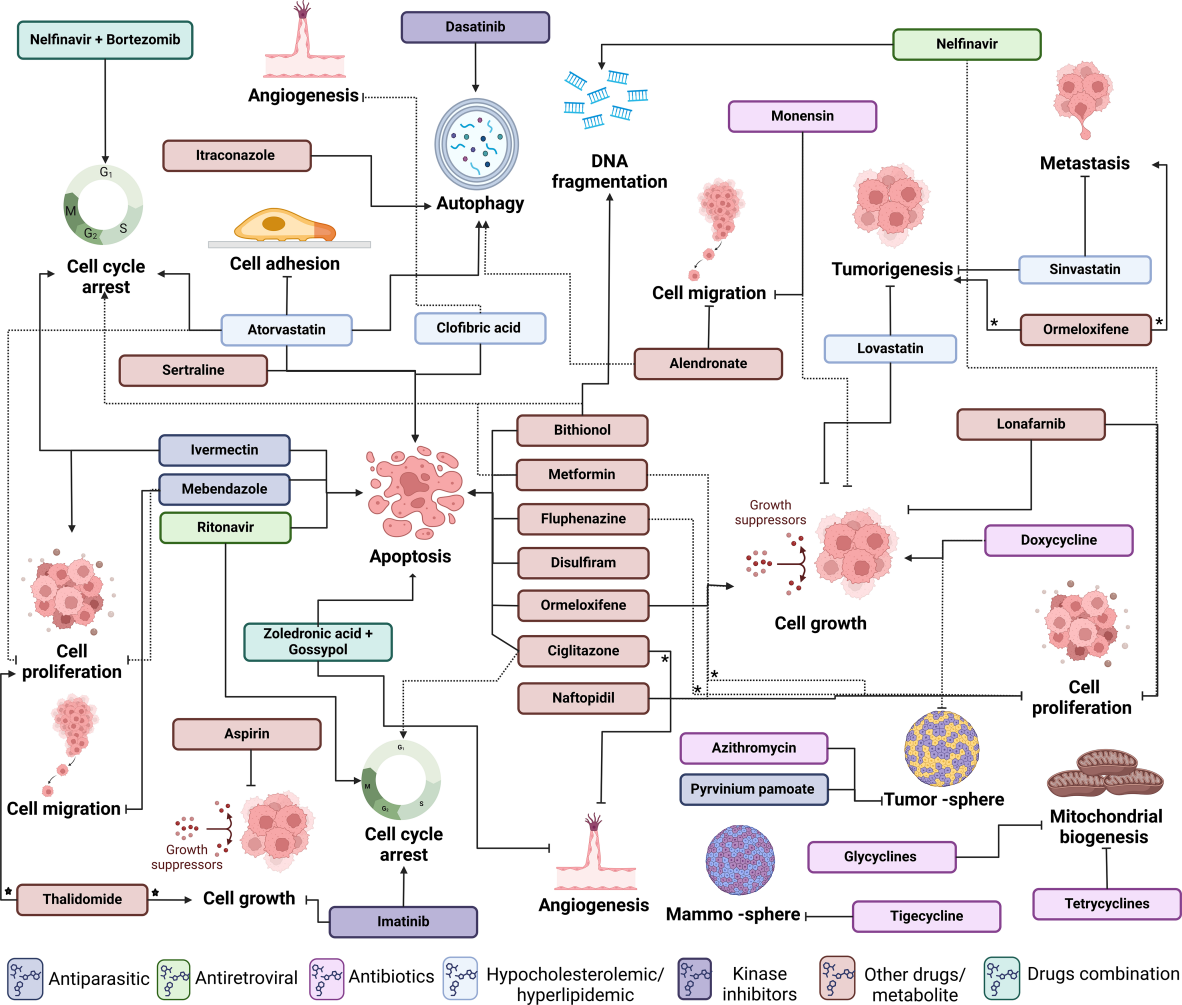
Cancer hallmarks involved in the drug reposition for ovarian cancer treatment. Schematic representation of the main metabolic pathways related to anti-tumor activities of repositioned drugs. The summary includes antiparasitic drugs (dark blue boxes), antiretroviral (light green boxes), antibiotics (grape boxes), compounds to treat hypocholesterolemia (light blue boxes), and other drugs and metabolites (red boxes). Compounds used in combinatorial treatments are also represented (dark green boxes). Induction (→), reduction (*), or inhibition (┤) of the cancer hallmarks is indicated within the figure (Figure created with Biorender).

Despite the enticing results from the *in silico* analyses, it is important to note that it is of the essence to perform subsequent *in vitro* and *in vivo* validations prior to clinical applications. However, computational approaches can provide information and further hypotheses for model-informed drug repurposing. As summarized in this review, **Figure 5** provides a schematic representation of central metabolic pathways associated with the anti-tumor activities of repurposed drugs. This overview encompasses antiparasitic drugs, antiretrovirals, antibiotics, hypocholesterolemia treatments, and other drugs and metabolites.

## Conclusion

This review provides valuable insights into the utility of drug repositioning for OC treatment, highlighting the importance of cell line and animal models in initial screening and drug testing. Although clinical translation remains in its early stages, these models form a crucial foundation for future studies. Overcoming the challenges of translating preclinical findings into successful human therapies will require careful consideration of drug interactions, personalized medicine approaches, and extensive clinical validation. We provided a critical discussion of the potential of drug repositioning for OC treatment, highlighting current advances in the area, which does not represent a clinical investigation. Our analysis focused on *in vitro* experiments across multiple cancer cell lines and short-term *in vivo* models, and the limited information available from clinical trials. The discussion revealed encouraging evidence of the efficacy of existing drugs in targeting OC. Despite these promising findings, we recognize the limitations inherent in *in vitro* and preclinical models. These systems, while informative, fail to capture the full complexity, heterogeneity, and microenvironment of OC in human patients, which limits the direct translation of findings to clinical practice. Key challenges include differences in pharmacokinetics and pharmacodynamics between preclinical models and human systems, which may influence therapeutic efficacy and safety profiles. Furthermore, *in vivo* and *in vitro* studies do not account for the complex pharmacological interactions that occur in real-world scenarios, such as those between drugs, dietary supplements, and food, which could either amplify or attenuate therapeutic effects. Careful optimization of dosing regimens is crucial to balance efficacy and minimize adverse outcomes. Looking forward, systematic approaches that integrate high-throughput screening, computational modeling, and patient-derived models offer a path to refining drug combinations and tailoring therapies to individual patients. These methodologies, combined with biomarker-driven approaches, enable the identification of molecular pathways most relevant to OC progression and therapeutic response. Leveraging genomics and proteomics can further clarify the pharmacokinetics and pharmacodynamics of repositioned drugs, supporting their clinical application.

Drug repositioning is particularly advantageous in oncology due to its ability to shorten the timeline for bringing effective therapies to patients, compared to the 10 - 15 years typically required for *de novo* drug development, as evidenced above. Despite the potential usage of a wide range of available drugs, the clinical validation of these therapies requires robust longitudinal studies and clinical trials to assess safety, efficacy, and optimal combination strategies in the context of OC. Ultimately, drug repositioning holds significant promise for overcoming challenges in OC treatment, particularly drug resistance. By addressing pharmacokinetic challenges, optimizing dosing strategies, and incorporating personalized medicine principles, repositioned drugs can offer cost-effective and innovative solutions. Nonetheless, clinical trials remain indispensable to substantiating preclinical findings and ensuring that repositioned therapies fulfill their potential in improving outcomes for OC patients. This review point to the critical role of multidisciplinary approaches in advancing the utility of drug repositioning for OC, with the ultimate goal of enhancing patient survival and quality of life.
